# Supplementary material for: Multicellular model of neuroblastoma proposes unconventional therapy based on multiple roles of p53
Source: PLoS Comput Biol. 2024 Dec 23;20(12):e1012648. doi: 10.1371/journal.pcbi.1012648 (PMC11723635; doi:10.1371/journal.pcbi.1012648)
Supplement: S1 Text — This text describes each model component in detail, including the continuous automaton, both agent types, the centre-based mechanical model, and the auxiliary functions linking them together in the overall multicellular model. (PDF) [file pcbi.1012648.s001.pdf]

S1 Text: Detailed Model Description

Kenneth Y. Wertheim[1,2,3,4], Robert Chisholm[2], Paul Richmond[2], Dawn Walker[1,2]

[1]Insigneo Institute for *in Silico* Medicine, University of Sheffield, Sheffield, UK.
[2]Department of Computer Science, University of Sheffield, Sheffield, UK.
[3]Centre of Excellence for Data Science, Artificial Intelligence, and Modelling, University of Hull, Kingston upon Hull, UK.
[4]School of Computer Science, University of Hull, Kingston upon Hull, UK.

This hybrid model of neuroblastoma comprises a continuous automaton describing the tumour microenvironment, an agent-based model describing the neuroblastoma and Schwann cells inhabiting this microenvironment, and a centre-based model of the cell-cell mechanical interactions within this microenvironment. Execution of this hybrid model involves a stochastic simulation, which comprises N_{steps} time steps lasting T_{step} (hours) each. Time is represented by t (hours), which goes from zero to $N_{steps} \times T_{step}$ hours.

Throughout this text, the variable *dummy* is used for various purposes, such as a flag or a counter. It has no biological meaning.

There are several systems-level homogeneous variables which are initialized at $t = 0$:

1. The dimensionless oxygen level, C_{O2}, is a continuous variable ranging from zero to one; the concentration scale is the oxygen concentration in the kidney, $C^s_{O2} = 72$ mmHg [1]. This variable may stay constant throughout a simulation or be updated at each time step, depending on a user-defined variable $static_{O2}$. If $static_{O2}$ is one, the assumption is that the vasculature always supplies enough oxygen to return C_{O2} to its equilibrium (initial value) at the end of each time step. Note that all the simulations referred to herein were conducted with $static_{O2}$ set to zero.

2. The oxygen supply rate, R_{O2}, is the increase in C_{O2} due to the vasculature in one time step, while N_{ang} is the integral number of angiogenic signals in the system. The integer N_{vf} is the number of VEGF-producing neuroblastoma cell agents in the entire continuous automaton.

3. The vector $chemo_{start}$ contains elements that are the time points at which chemotherapeutic cycles begin, while the elements in the vector $chemo_{end}$ are the time points at which the corresponding cycles end. The two vectors contain the same number of elements in agreement with the number of chemotherapeutic cycles in the simulation. The vector $chemo_{effects}$ describes the regimen's effects on neuroblastoma cell agents in different chemotherapeutic cycles, specifically their effects on CHK1, JAB1, HIF, MYCN, telomerase, and p53, which are gene products (proteins). Its elements are arranged in groups of six and the number of groups is the number of chemotherapeutic cycles. Each element is the probability that the corresponding protein is inhibited by chemotherapy in an hour during the corresponding period.

As shown in Fig 2 in the main article, the model is updated iteratively. In each iteration, the cell agents in a simulation are updated internally before the centre-based model (mechanical model henceforth) is solved numerically to equilibrate their positions. Finally, the continuous automaton representing the tumour microenvironment is updated. Further details of these model components are provided in the following sections.

A Continuous automaton

Like a cellular automaton, a continuous automaton uses a grid of voxels to represent space. Unlike a cellular automaton, the state of a voxel can have an infinite number of states [2].

Each voxel is a cube spanning L_{voxel} in each dimension. Therefore, we can label a voxel with the vector $voxel = (i, j, k)$; i, j, and k describe where the voxel is in the domain. Initially, the central voxel is located at the centre of the virtual tumour, and in each dimension, there are as many voxels in the negative direction of the central voxel as there are in its positive direction. It follows that initially there is an odd number of voxels in each dimension. The 3D von Neumann neighbourhood of the voxel denoted by (i, j, k) includes itself and the six voxels immediately adjacent to it: (i+1, j, k), (i-1, j, k), (i, j+1, k), (i, j-1, k), (i, j, k+1), and (i, j, k-1). The total number of voxels in the continuous automaton is denoted by N_{vox}.

Each voxel is associated with a state vector, $voxstate_{i,j,k} = (N_{i,j,k}, N^a_{i,j,k}, N^n_{i,j,k}, N^l_{i,j,k}, S_{i,j,k}, S^a_{i,j,k}, S^n_{i,j,k}, S^l_{i,j,k}, S^{lm}_{i,j,k}, NS_{i,j,k}, M_{i,j,k})$; the first four elements are the numbers of neuroblastoma cells (total), apoptotic neuroblastoma cells, necrotic neuroblastoma cells, and living neuroblastoma cells respectively; the next five are the numbers of Schwann cells (total), apoptotic Schwann cells, necrotic Schwann cells, living Schwann cells, and matrix-producing Schwann cells respectively; the last two are the total number of cells ($N_{i,j,k} + S_{i,j,k}$) and the fraction of volume occupied by extracellular matrix. All but $M_{i,j,k}$ are non-negative integers, while $M_{i,j,k}$ is continuous and ranges from zero to one.

As shown in Fig 2 in the main article, the continuous automaton is updated after the two agent populations and mechanical model.

if cell agents exist beyond the continuous automaton **then**
  add voxels in all three dimensions
  matrix volume fraction in new voxels ← average matrix volume fraction in old voxels
  update N_{vf}
  update $voxstate_{i,j,k}$ for all (i, j, k)
else
  update N_{vf}
  update $voxstate_{i,j,k}$ for all (i, j, k)
end if

B Neuroblastoma cell agents

Each neuroblastoma cell agent's state is defined by four types of attributes, which are recorded in four vectors: physical, cellular, mutation, and molecular vectors. The agent is denoted by the index n.

1. Physical vector: $physical_n = (x_n, y_n, z_n, F_{x,n}, F_{y,n}, F_{z,n}, \delta^{overall}_n, N_{nghbr,n}, mobile_n)$. These are the agent's spatial coordinates in microns, the net forces acting on it in the three spatial dimensions (N), its overlap with the other agents in the system (only positive and in microns), the number of agents in its neighbourhood (including itself), and a Boolean variable indicating whether it is mobile and can generate a locomotive force.

2. Cellular vector: $cellular_n = (cycle_n, apop_n, signal_{apop,n}, necro_n, signal_{necro,n}, signal_{necro,c}, N_{telo,n}, deg_{diff,n}, P^0_{cyc,n}, hypoxia_n, nutrient_n, DNA_{damage,n}, DNA_{unrep,n})$. $cycle_n$ is a continuous variable labelling the agent's position in the cell cycle (zero to four; with zero indicating G0, one indicating G1/S, two indicating S/G2, three indicating G2/M, and four indicating division [3]). $apop_n$ is a Boolean variable indicating whether the agent is apoptotic. $signal_{apop,n}$ is a discrete variable representing the number of apoptotic signals the agent has. The time from the initiation of apoptosis to its completion is as short as two or three hours [4]. $necro_n$ is a Boolean variable indicating whether the agent is necrotic. $signal_{necro,n}$ is a discrete variable representing the number of necrotic signals the agent has. $signal_{necro,c}$ is a discrete variable representing the number of necrotic signals necessary for it to turn necrotic (necrosis occurs over prolonged periods from a few hours to approximately one week [5]). $N_{telo,n}$ is a discrete variable representing its telomere lengths abstractly (a normal human foetal cell can divide between 40 and 60 times before reaching the Hayflick limit and entering a senescence phase due to shortening telomeres [6]). $deg_{diff,n}$ is a continuous variable ranging from zero to one that reflects its extent of differentiation. $P^0_{cyc,n}$ is a continuous variable ranging from zero to one that models the probability that the agent can re-enter the cell cycle after division (complement of $deg_{diff,n}$). $hypoxia_n$ is a Boolean variable indicating whether the agent is hypoxic, while $nutrient_n$ is another Boolean variable indicating whether it has

access to enough nutrients. $DNA_{damage,n}$ is a Boolean variable indicating whether the agent has damaged DNA and $DNA_{unrep,n}$ is a Boolean variable indicating whether it has unreplicated DNA.

3. Mutation vector: $mutation_n = (MA_n, TR_n, AI_n, ALK_n)$. The four elements indicate whether the agent's *MYCN* gene is amplified, whether its *TERT* gene is rearranged, whether its *ATRX* gene is inactivated, and the status of its *ALK* gene respectively. The first three elements are Boolean variables. The last element is a discrete variable indicating *ALK*'s mutation status: none (0), amplified or activated (1), and other *RAS* mutations (2).

4. Molecular vector: $molecular_n = (ATP_n, telo_n, ALT_n, MYCN_n, MR_n, JAB1_n, CHK1_n, CDS1_n, CDC25C_n, ID2_n, IAP2_n, HIF_n, BNIP3_n, VEGF_n, p53_n, p73_n, p21_n, p27_n, Bcl_n, BAK/BAX_n, CAS_n)$. ATP_n is a Boolean variable indicating whether the agent has sufficient ATP. $telo_n$ is a Boolean variable indicating whether telomerase is active in the agent. ALT_n is a Boolean variable indicating if alternative lengthening of telomeres is active therein. The remaining vector elements are Boolean variables indicating whether the denoted species are active therein.

During a time step, the following transition functions are used to update the state vectors: *Sense*, *Cycle*, *Divide* and *Remove*.

B.1 Sense

This function represents the tumour's microenvironment's influence on the neuroblastoma cell represented by an agent. It changes the agent's intracellular conditions to influence its phenotypic behaviours (subsequent transition functions). Unless otherwise stated, the source of this subsection is a book chapter [3]. These are the specific updates encapsulated within this function.

1. Responses to stressors: hypoxia and lack of glucose.
2. Changes to DNA.
3. Responses to necrotic signals.
4. Intracellular signalling.
5. Intercellular signalling.
6. Responses to apoptotic signals.
7. Apoptosis and necrosis.

There are two major stressors. First, as in [5], hypoxia-induced necrosis is a saturable process in our model and the probability of hypoxia is $1 - \frac{C^s_{O2} \times C_{O2}}{C^s_{O2} \times C_{O2} + C^{50}_{O2}}$. Second, the cell agent requires other nutrients, such as glucose. Oxygen availability is considered a sufficient proxy for resource availability [5], partly because oxygen and other resources are collectively distributed by the vasculature, so their concentration profiles should be highly correlated. Unless otherwise stated, every random number (represented by *rand*) is a sample of the uniform distribution between 0 and 1.

if $1 - \frac{C^s_{O2} \times C_{O2}}{C^s_{O2} \times C_{O2} + C^{50}_{O2}} > rand$ **then**
  $hypoxia_n \leftarrow 1$
else
  $hypoxia_n \leftarrow 0$
end if

if $1 - \frac{C^s_{O2} \times C_{O2}}{C^s_{O2} \times C_{O2} + C^{50}_{O2}} > rand$ **then**
  $nutrient_n \leftarrow 0$
else
  $nutrient_n \leftarrow 1$
end if

An agent's DNA changes due to these stressors, but it may also get repaired by p53 [7] and p73 [8].

if $DNA_{damage,n} == 1$ AND ($p53_n == 1$ OR $p73_n == 1$) **then**
  $DNA_{damage,n} \leftarrow 0$
end if

DNA damage is related to shortening telomeres. As stated above, a normal human foetal cell can divide between 40 and 60 times before reaching the Hayflick limit and entering a senescence phase due to shortening telomeres [6]. After 40 divisions, it will become increasingly susceptible to DNA damage due to its shortening telomeres. In the absence of any telomere repair mechanisms (telomerase or ALT pathway), the neuroblastoma cell agent's probability of having damaged DNA will become 100 % after 60 divisions. Therefore, the probability of having its DNA damaged in an hour is given by $1 - \frac{N_{telo,n}}{N_{telo,c}}$ [6].

if $DNA_{damage,n} == 0$ AND $(1 - \frac{N_{telo,n}}{N_{telo,c}}) \times T_{step} > rand$ **then**
  $DNA_{damage,n} \leftarrow 1$
end if

In our model, an agent with damaged DNA is susceptible to apoptosis, but it is acknowledged that after reaching the Hayflick limit, a real cell becomes senescent: it stops cycling, but does not always die [9]. However, like apoptotic cells, senescent cells are engulfed by the immune system [10]. For the sake of simplicity, senescent cell agents are considered to have damaged DNA (apoptotic).

Independently of the Hayflick limit, the chance that the agent's DNA is damaged is increased during hypoxia [11].

if $DNA_{damage,n} == 0$ AND $hypoxia_n == 1$ AND $P_{DNA,h} \times T_{step} > rand$ **then**
  $DNA_{damage,n} \leftarrow 1$
end if

Finally, independently of the Hayflick limit and hypoxia, chemotherapy can damage the agent's DNA if the agent is in the S phase of the cell cycle.

if $\exists i : chemo_{start,i} <= t <= chemo_{end,i}$ AND $mean(chemo_{effects}) > rand$ **then**
  if $DNA_{damage,n} == 0$ AND $1 < cycle_n < 2$ AND $P_{DNA,c} \times T_{step} > rand$ **then**
    $DNA_{damage,n} \leftarrow 1$
  end if
end if

Two potentially additive sources of necrotic signals are considered. They are additive in the sense that the two triggers are not mutually exclusive and can co-occur in one time step.

Necrotic cells release their contents in an uncontrolled manner, including microbial damaging substances. These substances can damage living cells, leading to inflammation [12]. In a living agent's 3D von Neumann neighbourhood, a necrotic cell agent can induce necrosis in the living agent.

The second trigger for necrosis depends on the availability of ATP and how it is generated. When there is enough glucose but not enough oxygen, ATP is generated by glycolysis, which releases lactic acid and is approximately less effective than oxidative phosphorylation by a factor of 15 [13].

After considering both sources, if the cell agent does not have at least one new necrotic signal ($stress$ is zero), it may lose some of its necrotic signals (recovery).

$dummy \leftarrow 0$
$stress \leftarrow 0$

while $dummy < \sum_{3DVNN}(N^n_{i,j,k} + S^n_{i,j,k})$ **do**
  if $P^{is}_{necro} \times T_{step} > rand$ **then**
    increase $signal_{necro,n}$ by T_{step}
    $stress \leftarrow 1$

    end if
    increase $dummy$ by one
end while

if $nutrient_n == 1$ **then**
    if $hypoxia_n == 0$ **then**
        $ATP_n \leftarrow 1$
        if $signal_{necro,n} > 0$ AND $stress == 0$ AND $P_{necro,r} \times T_{step} > rand$ **then**
            reduce $signal_{necro,n}$ by T_{step}
        end if
    else
        if $R_{glycolysis} > rand$ **then**
            $ATP_n \leftarrow 1$
            increase $signal_{necro,n}$ by T_{step}
        else
            $ATP_n \leftarrow 0$
            increase $signal_{necro,n}$ by $2 \times T_{step}$
        end if
    end if
else
    $ATP_n \leftarrow 0$
    increase $signal_{necro,n}$ by T_{step}
end if

In the fourth part of the *Sense* function, the gene products are evaluated one by one in the following order to mimic intracellular signalling.

The Boolean variables $MYCN_n$, MR_n, and $JAB1_n$ are set to zero or one according to their respective Bernoulli trials, parameterised by the probabilities $P_{MYCN,n}$, $P_{MR,n}$, and $P_{JAB1,n}$ respectively. Subsequently, the effects of chemotherapy on MYCN (fourth element of $chemo_{effects}$) and JAB1 (second element of $chemo_{effects}$) are represented in the following two Bernoulli trials.

if $MYCN_n == 1$ AND $\exists i : chemo_{start,i} <= t <= chemo_{end,i}$ AND $chemo_{effects,4} > rand$ **then**
    $MYCN_n \leftarrow 0$
end if

if $JAB1_n == 1$ AND $\exists i : chemo_{start,i} <= t <= chemo_{end,i}$ AND $chemo_{effects,2} > rand$ **then**
    $JAB1_n \leftarrow 0$
end if

CHK1 is then evaluated. The p53-independent mechanism of cell cycle arrest triggered by damaged or unreplicated DNA involves Rad proteins. They activate CDS1 in response to unreplicated DNA and CHK1 in response to damaged DNA (G2 DNA damage response). MYCN upregulates CHK1 [14]. Both CDS1 and CHK1 phosphorylate (inhibit) CDC25C to create a binding site for the 14-3-3 family of phosphoserine binding proteins; the resulting binding event sequesters CDC25C to the cytoplasm, thereby preventing it from interacting with cyclin B/CDC2, which is localised to the nucleus between G2 and M. These interactions are represented by the following conditional statements, where $P_{CHK1,n}$ is the probability of success in a Bernoulli trial.

if $DNA_{damage,n} == 1$ AND $P_{CHK1,n} > rand$ **then**
    $CHK1_n \leftarrow 1$
else
    if $DNA_{damage,n} == 1$ AND $P_{CHK1,n} > rand$ AND $MYCN_n == 1$ **then**
        $CHK1_n \leftarrow 1$
    else
        $CHK1_n \leftarrow 0$
    end if

end if

CHK1 is a target of chemotherapy (first element of $chemo_{effects}$). Its inhibition is modelled by the following conditional statement.

if $CHK1_n == 1$ AND $\exists i : chemo_{start,i} <= t <= chemo_{end,i}$ AND $chemo_{effects,1} > rand$ **then**
  $CHK1_n \leftarrow 0$
end if

The next species of interest is ID2, which is activated by MYCN [14]. The corresponding Bernoulli trial is parameterised by $P_{ID2,n}$.

if $MYCN_n == 1$ AND $P_{ID2,n} > rand$ **then**
  $ID2_n \leftarrow 1$
else
  $ID2_n \leftarrow 0$
end if

The next species is IAP2, whose increased expression is induced by the hypoxia-induced transcription factor called nuclear factor kappa B; this is independent of HIF-1 [15]. The corresponding Bernoulli trial is parameterised by $P_{IAP2,n}$.

if $hypoxia_n == 1$ AND $P_{IAP2,n} > rand$ **then**
  $IAP2_n \leftarrow 1$
else
  $IAP2_n \leftarrow 0$
end if

Next, the function will evaluate HIF-1, which is induced by hypoxia at the protein level [15]. JAB1 and p53 bind competitively to the same domain on HIF-1α to stabilise and degrade it respectively [16]. In addition, p73 promotes its degradation [17]. These positive and negative interactions are represented by two separate groups of conditional statements, including Bernoulli trials parameterised by $P_{HIF,n}$.

if $hypoxia_n == 1$ AND $P_{HIF,n} > rand$ **then**
  $HIF_n \leftarrow 1$
else
  if $hypoxia_n == 1$ AND $P_{HIF,n} > rand$ AND $JAB1_n == 1$ **then**
    $HIF_n \leftarrow 1$
  else
    $HIF_n \leftarrow 0$
  end if
end if

if $HIF_n == 1$ AND ($p53_n == 1$ OR $p73_n == 1$) **then**
  $HIF_n \leftarrow 0$
end if

In addition, HIF is a target of chemotherapy (third element of $chemo_{effects}$).

if $HIF_n == 1$ AND $\exists i : chemo_{start,i} <= t <= chemo_{end,i}$ AND $chemo_{effects,3} > rand$ **then**
  $HIF_n \leftarrow 0$
end if

BNIP3 mediates apoptosis via HIF-1. Cells lacking HIF-1 cannot produce BNIP3 abundantly. The BNIP3 promoter contains a hypoxia response element (HRE), so HIF-1 can induce the expression of this gene [15]. The Bernoulli trial regulating BNIP3 is parameterised by $P_{BNIP3,n}$.

if $HIF_n == 1$ AND $P_{BNIP3,n} > rand$ **then**
  $BNIP3_n \leftarrow 1$
else
  $BNIP3_n \leftarrow 0$
end if

VEGF is the next species. HIF-1α and HIF-2α are involved throughout the process of blood vessel formation [11]. First, they upregulate VEGF to recruit endothelial progenitor cells from bone marrow and induce their differentiation into endothelial cells. Second, they upregulate pro-angiogenic molecules such as VEGF-R2, FGF family members, and PDGF. Third, they upregulate MMPs, VEGF-R1, Ang-1, and Ang-2 to promote cell migration. Finally, they upregulate Ang-1, PDGF, and TGF-β to recruit supporting cells such as smooth muscle cells and pericytes to support vessel maturation. For the sake of simplicity, these effects are combined into one Bernoulli trial representing VEGF expression, parameterised by $P_{VEGF,n}$.

if $HIF_n == 1$ AND $P_{VEGF,n} > rand$ **then**
  $VEGF_n \leftarrow 1$
else
  $VEGF_n \leftarrow 0$
end if

The next species, p53, is active in multiple scenarios.

1. The presence of damaged DNA triggers immunoprecipitated ATM to phosphorylate p53 on the residue Ser15 *in vivo*, causing it to dissociate from MDM2 and stabilise as a result. For example, dysfunctional telomeres limit tumorigenesis by activating p53-dependent cellular senescence and apoptosis [18].
2. HIF-1α stabilises p53 by binding to MDM2 [19].
3. MYCN can both upregulate and downregulate p53 depending on the context [14, 20]. According to a study [20], its expression at a high level is associated with favourable clinical outcomes in the absence of *MYCN* amplification, but it bodes ill when this mutation is present. The *p53* gene is a direct transcriptional target of MYCN in neuroblastoma: a likely mechanism for MYCN-induced p53-dependent apoptosis [21]. However, *MYCN*-amplified neuroblastoma cells may circumvent the mechanism in the presence of aberrations in the p53/MDM2/ARF pathway [21]. In fact, mutations in the *p53* gene are rare at diagnosis, so MYCN likely affects p53 suppressors, such as MDM2 [14]. By contrast, mutations in *p53* itself are common at relapse [14].

These scenarios are represented by the following conditional statements, which are parameterised by $P_{p53,n}$.

if $DNA_{damage,n} == 1$ AND $P_{p53,n} > rand$ **then**
  $p53_n \leftarrow 1$
else
  if $DNA_{damage,n} == 1$ AND $MYCN_n == 1$ AND $P_{p53,n} > rand$ **then**
    $p53_n \leftarrow 1$
  else
    if $HIF_n == 1$ AND $P_{p53,n} > rand$ **then**
      $p53_n \leftarrow 1$
    else
      if $HIF_n == 1$ AND $MYCN_n == 1$ AND $P_{p53,n} > rand$ **then**
        $p53_n \leftarrow 1$
      else
        $p53_n \leftarrow 0$
      end if
    end if
  end if

end if

After the above steps, the function will implement an abstract representation of MYCN's association with poor clinical outcomes when *MYCN* is amplified. This is necessary because the p53/MDM2/ARF pathway is not explicitly included in our model.

if $p53_n == 1$ AND $MYCN_n == 1$ AND $MA_n == 1$ **then**
  $p53_n \leftarrow 0$
end if

p53 is a target of chemotherapy (sixth element of $chemo_{effects}$).

if $p53_n == 1$ AND $\exists i : chemo_{start,i} <= t <= chemo_{end,i}$ AND $chemo_{effects,6} > rand$ **then**
  $p53_n \leftarrow 0$
end if

p73 is the next protein to be evaluated. Dysfunctional telomeres activate an ATR-CHK1-dependent DNA damage response to initiate a robust p53-independent, p73-dependent apoptotic pathway [18]. Hypoxia, via HIF-1α, stabilises p73 too [22]. These mechanisms are parameterised by $P_{p73,n}$.

if $CHK1_n == 1$ AND $P_{p73,n} > rand$ **then**
  $p73_n \leftarrow 1$
else
  if $HIF_n == 1$ AND $P_{p73,n} > rand$ **then**
    $p73_n \leftarrow 1$
  else
    $p73_n \leftarrow 0$
  end if
end if

The next two species share the following regulatory mechanisms. First, hypoxia upregulates both p21 and p27 via HIF-1α [23]. Second, damaged DNA upregulates both species through p53-mediated transcriptional activation [24, 25]. Third, MAPK/RAS signalling suppresses both p21 and p27 [26]. Fourth, MYCN downregulates both p21 [27] and p27 [28].

The two activating mechanisms are parameterised by $P_{p21,n}$ and $P_{p27,n}$.

if $HIF_n == 1$ AND $P_{p21,n} > rand$ **then**
  $p21_n \leftarrow 1$
else
  if $p53_n == 1$ AND $P_{p21,n} > rand$ **then**
    $p21_n \leftarrow 1$
  else
    $p21_n \leftarrow 0$
  end if
end if

if $HIF_n == 1$ AND $P_{p27,n} > rand$ **then**
  $p27_n \leftarrow 1$
else
  if $p53_n == 1$ AND $P_{p27,n} > rand$ **then**
    $p27_n \leftarrow 1$
  else
    $p27_n \leftarrow 0$
  end if
end if

The two suppressive mechanisms are represented by the following conditional statements.

if $p21_n == 1$ AND $MR_n == 1$ **then**
  $p21_n \leftarrow 0$
else
  if $p21_n == 1$ AND $MYCN_n == 1$ **then**
    $p21_n \leftarrow 0$
  end if
end if

if $p27_n == 1$ AND $MR_n == 1$ **then**
  $p27_n \leftarrow 0$
else
  if $p27_n == 1$ AND $MYCN_n == 1$ **then**
    $p27_n \leftarrow 0$
  end if
end if

Bcl-2 and Bcl-xL are anti-apoptotic proteins. They are represented by one abstract entity in our model. BNIP3 binds to and inhibits both species [15]. Acting via GRAMD4, p53 and p73 have an inhibitory effect on them too [29]. The abstract entity is regulated by a Bernoulli trial parameterised by $P_{Bcl,n}$ and a conditional statement.

if $P_{Bcl,n} > rand$ **then**
  $Bcl_n \leftarrow 1$
else
  $Bcl_n \leftarrow 0$
end if

if $Bcl_n == 1$ AND ($BNIP3_n == 1$ OR $p53_n == 1$ OR $p73_n == 1$) **then**
  $Bcl_n \leftarrow 0$
end if

BAX and BAK are also represented by one abstract entity. There are three pathways towards its activation. p53 activates both species, while p73 activates BAX [29]. Hypoxia inhibits the electron transport chain on the inner membrane of a mitochondrion, thus reducing the amount of ATP that can be derived from it; BAX and BAK are activated as a result to induce apoptosis [15]. There are two suppressive mechanisms. IAP2 prevents BAX from translocating to the mitochondria to induce apoptosis [15]. Bcl-2 and Bcl-xL suppress both BAX and BAK [15].

The conditional statements modelling the activating mechanisms are parameterised by $P_{BAX/BAK,n}$.

if $hypoxia_n == 1$ AND $P_{BAX/BAK,n} > rand$ **then**
  $BAX/BAK_n \leftarrow 1$
else
  if $p53_n == 1$ AND $P_{BAX/BAK,n} > rand$ **then**
    $BAX/BAK_n \leftarrow 1$
  else
    if $p73_n == 1$ AND $P_{BAX/BAK,n} > rand$ **then**
      $BAX/BAK_n \leftarrow 1$
    else
      $BAX/BAK_n \leftarrow 0$
    end if
  end if
end if

Their suppressive mechanisms are modelled by a conditional statement too.

if $BAX/BAKn == 1$ AND $(Bcl_n == 1$ OR $IAP2_n == 1)$ **then**
  $BAX/BAK_n \leftarrow 0$
end if

CAS is another abstract entity representing the collection of caspases in a neuroblastoma cell. It has two activating mechanisms. First, BAX and BAK cause cytochrome C to be released from a mitochondrion into the cytoplasm to initiate the apoptotic cascade, which activates caspase 9 via Apaf-1 to cleave caspases 3 and 6, ultimately resulting in cell death [15]. Second, hypoxia generates radicals like ROS to trigger an activation cascade by which caspase 9 is cleaved directly by caspases 3 and 12 [15]. Separately, hypoxia activates JNK to switch on caspases [15]. Since ROS and JNK are not in the model, a direct link between hypoxia and CAS is used to model the second activating mechanism. Both mechanisms are energy-dependent processes [15]. The conditional statements representing them are parameterised by $P_{CAS,n}$.

if $BAX/BAK_n == 1$ AND $ATP_n == 1$ AND $P_{CAS,n} > rand$ **then**
  $CAS_n \leftarrow 1$
else
  if $hypoxia_n == 1$ AND $ATP_n == 1$ AND $P_{CAS,n} > rand$ **then**
    $CAS_n \leftarrow 1$
  else
    $CAS_n \leftarrow 0$
  end if
end if

Unreplicated DNA repair is then attempted. It is known that p53 [7] and p73 [8] are involved in repairing unreplicated DNA. This repair mechanism is modelled by the following conditional statement.

if $DNA_{unrep,n} == 1$ AND $(p53_n == 1$ OR $p73_n == 1)$ **then**
  $DNA_{unrep,n} \leftarrow 0$
end if

The final two species to be evaluated are CDS1 and CDC25C. Rad proteins are involved in the p53-independent mechanism of cell cycle arrest triggered by damaged or unreplicated DNA. They activate CDS1 in response to unreplicated DNA and CHK1 in response to damaged DNA (G2 DNA damage response). MYCN upregulates CHK1 [14]. Both CDS1 and CHK1 phosphorylate (inhibit) CDC25C to create a binding site for the 14-3-3 family of phosphoserine binding proteins; the resulting binding event sequesters CDC25C to the cytoplasm, thereby preventing its interactions with cyclin B/CDC2, which is localised to the nucleus between G2 and M.

Their activating mechanisms are parameterised by $P_{CDS1,n}$ and $P_{CDC25C,n}$ in the following conditional statements.

if $DNA_{unrep,n} == 1$ AND $P_{CDS1,n} > rand$ **then**
  $CDS1_n \leftarrow 1$
else
  $CDS1_n \leftarrow 0$
end if

if $P_{CDC25C,n} > rand$ **then**
  $CDC25C_n \leftarrow 1$
else
  $CDC25C_n \leftarrow 0$
end if

Only CDC25C has suppressive mechanisms, which are modelled by the following conditional statements.

if $CDC25C_n == 1$ AND $CDS1_n == 1$ **then**
  $CDC25C_n \leftarrow 0$
else
  if $CDC25C_n == 1$ AND $CHK1_n == 1$ **then**
    $CDC25C_n \leftarrow 0$
  end if
end if

After these intracellular signalling events, the *Sense* function integrates the juxtacrine and paracrine signals the agent receives from its neighbours. The key metric for juxatacrine signalling is the ratio of the number of living Schwann cell agents to the number of living cell agents in this agent's 3D von Neumann neighbourhood: $ratio_{3DVNN} = \frac{\sum_{3DVNN} S^l_{i,j,k}}{\sum_{3DVNN} N^l_{i,j,k} + \sum_{3DVNN} S^l_{i,j,k}}$. The key metric for paracrine signalling is the ratio of the number of living Schwann cell agents to the number of living cell agents in the entire continuous automaton: $ratio_{CA} = \frac{\sum_{CA} S^l_{i,j,k}}{\sum_{CA} N^l_{i,j,k} + \sum_{CA} S^l_{i,j,k}}$.

The following two Bernoulli trials model the ability of Schwann cells to promote differentiation in neuroblastoma cells by juxtacrine and paracrine signalling respectively [30].

if $R^{jux}_{diff,nb} \times ratio_{3DVNN} \times T_{step} > rand$ **then**
  $deg_{diff,n} \leftarrow min\{deg_{diff,n} + R_{diff} \times T_{step}, 1\}$
else
  if $R^{para}_{diff,nb} \times ratio_{CA} \times T_{step} > rand$ **then**
    $deg_{diff,n} \leftarrow min\{deg_{diff,n} + R_{diff} \times T_{step}, 1\}$
  end if
end if

Cell cycling ability is related to the extent of differentiation. Usually, more differentiated cells are less proliferative [31]. Therefore, the *Sense* function will update $P^0_{cyc,n}$ by setting it to $1 - deg_{diff,n}$.

The agent's number of apoptotic signals will be updated next. There are multiple triggers for a neuroblastoma cell to acquire apoptotic signals, including CAS, damaged DNA, and Schwann cells [30]. The following conditional statements represent these mechanisms in our model.

$stress \leftarrow 0$
if $CAS_n == 1$ **then**
  $signal_{apop,n} \leftarrow signal_{apop,n} + T_{step}$
  $stress \leftarrow 1$
else
  if $DNA_{damage,n} == 1$ AND $ATP_n == 1$ AND $P_{apop} * T_{step} > rand$ **then**
    $signal_{apop,n} \leftarrow signal_{apop,n} + T_{step}$
    $stress \leftarrow 1$
  end if
end if

if $R^{jux}_{apop,nb} \times ratio_{3DVNN} \times T_{step} > rand$ **then**
  $signal_{apop,n} \leftarrow signal_{apop,n} + T_{step}$
  $stress \leftarrow 1$
else
  if $R^{para}_{apop,nb} \times ratio_{CA} \times T_{step} > rand$ **then**
    $signal_{apop,n} \leftarrow signal_{apop,n} + T_{step}$
    $stress \leftarrow 1$
  end if
end if

If the above mechanisms are not active, the neuroblastoma cell agent will lose apoptotic signals. This process is modelled by the following conditional statement.

if $stress == 0$ AND $signal_{apop,n} > 0$ AND $P_{apop,r} \times T_{step} > rand$ **then**
    $signal_{apop,n} \leftarrow signal_{apop,n} - T_{step}$
end if

The *Sense* function ends with the agent's apoptotic status and necrotic status. Apoptotic cells in any system die and disappear relatively quickly, as quickly as two or three hours after initiation [4]. As for necrosis, hypoxia results in necrosis over a prolonged period ranging from a few hours to approximately one week [5]. In addition, an apoptotic cell may undergo secondary necrosis [32]. Intuitively, an apoptotic or necrotic cell has inactive telomere repair mechanisms and intracellular signalling species. It is also immobile and has no ATP. The following conditional statements are used to implement these updates.

if $signal_{apop,n} >= T_{apop}$ **then**
    $mobile_n \leftarrow 0$
    $apop_n \leftarrow 1$
    set every element in the agent's molecular vector to zero
else
    if $signal_{necro,n} >= signal_{necro,c}$ **then**
        $mobile_n \leftarrow 0$
        $necro_n \leftarrow 1$
        set every element in the agent's molecular vector to zero
    else
        if $apop_n == 1$ AND $necro_n == 0$ AND $P_{necro,2} \times T_{step} > rand$ **then**
            $apop_n \leftarrow 0$
            $necro_n \leftarrow 1$
        else
            $apop_n \leftarrow apop_n$
            $necro_n \leftarrow necro_n$
        end if
    end if
end if

B.2 Cycle

This function models a neuroblastoma cell agent's progress through the cell cycle. Unless otherwise stated, the source of this subsection is a book chapter [3].

A neuroblastoma cell agent can only progress in the cell cycle if all of the following conditions are met.

1. It must not be inhibited by its immediate neighbours (contact inhibition) [33].
2. It must have enough ATP to meet the energy requirement of cell cycling [33].
3. By intuition, only a living cell can cycle, so it must be neither apoptotic nor necrotic.

The above conditions are checked by the following conditional statements.

if $N_{nghbr,n} <= N_{nghbr,max}$ AND $ATP_n == 1$ AND $apop_n == 0$ AND $necro_n == 0$ **then**
    if $P_{cycle,nb} > rand$ **then**
        do nothing
    else
        exit *Cycle* function
    end if
else
    exit *Cycle* function
end if

The cell cycle comprises distinct phases: G0 (quiescent or resting), G1, S, G2 and M. The non-quiescent phases last 12 (T_{G1}), six (T_S), four (T_{G2}), and two (T_M) hours respectively. What is not stated in the article is that an agent's radius increases from $\frac{L_{cell}}{2}$ to L_{cell} as it progresses through G1 and G2 of the cell

cycle. The increase in its radius is split into 16 parts corresponding to the 12 and four hours constituting G1 and G2 respectively. For example, during G1, its radius is $\frac{L_{cell}}{2} + \frac{L_{cell}}{2} \times \frac{12 \times cycle_n}{16}$.

The cell enters the G0 phase ($cycle_n$ is zero) immediately after dividing. A more differentiated cell is less proliferative and less likely to re-enter the cell cycle by exiting G0 to enter the G1 phase ($0 < cycle_n < 1$) [31]. There are two independent mechanisms that trigger this transition. The first involves RAS-dependent activation of the MAPK cascade (MAPK/RAS signalling henceforth) or MYCN. p21 and p27 must be inactive for this mechanism to work. In the second mechanism, ID2 triggers the transition.

In the early stage of G1, cyclin D/CDK4 and cyclin D/CDK6 are needed for cell cycle progression. They phosphorylate Rb pocket proteins, which repress the E2F transcription factors that transcribe the genes necessary for entry into the S phase. MAPK/RAS signalling activates transcription factors to transactivate the promoter of cyclin D, activating cyclin D/CDK4 and cyclin D/CDK6 as a result. Like MAPK/RAS signalling, MYCN upregulates cyclin D2 [28]. It also upregulates both CDK4 and SKP2, allowing CDK2 to escape inhibition by p21 [14]. p21, a CDKI, inhibits various G1 cyclin/CDK complexes, mainly cyclin/CDK2 complexes [34], but p21 can effectively inhibit CDK2, CDK3, CDK4, and CDK6 too [35]. p27 (another CDKI) also inhibits cyclin D/CDK4 [36] and CDK2 [37]. Therefore, p21 and p27 must be inactive.

The second pathway is independent of MAPK/RAS signalling, p21, and p27. It only requires that ID2 be active. ID2 inactivates Rb, thus activating E2F [14].

This transition from G0 to G1 is represented by the following conditional statements, which apply when $cycle_n$ is zero.

if $P^0_{cyc,n} > rand$ **then**
  if ($MR_n == 1$ OR $MYCN_n == 1$) AND $p21_n == 0$ AND $p27_n == 0$ **then**
    $cycle_n \leftarrow cycle_n + \frac{T_{step}}{T_{G1}}$
  else
    if $ID2_n == 1$ **then**
      $cycle_n \leftarrow cycle_n + \frac{T_{step}}{T_{G1}}$
    else
      $cycle_n \leftarrow 0$
    end if
  end if
else
  $cycle_n \leftarrow 0$
end if

The early stages of G1 are governed by the same two independent mechanisms involving MAPK/RAS signalling, MYCN, p21, p27, and ID2. However, the agent has already re-entered the cell cycle, so its degree of differentiation is not evaluated in this part of the *Cycle* function.

At the end of G1, there is a cell cycle checkpoint (restriction point). In the late stage of G1, cyclin E/CDK2 is necessary for initiating DNA replication and hence S phase entry. Like cyclin D/CDK4 and cyclin D/CDK6, this cyclin complex inhibits Rb by phosphorylation, which releases its grip on E2F, the transcription factor necessary for passage through the restriction point. MAPK/RAS signalling upregulates the CDKIs involved in the assembly of cyclin A/CDK2 and cyclin E/CDK2. Unlike the case of cyclin D, MYCN cannot compensate for this function of MAPK/RAS signalling, so MAPK/RAS signalling is needed continuously throughout G1 until the restriction point [38]. If interrupted, the cell will exit to G0 immediately, but upon re-stimulation, it will return to its current point in G1.

The agent's transition from G1 to the S phase ($1 \leq cycle_n < 2$) is represented by the following conditional statements, which are implemented every time after it makes progress in the G1 phase.

if $cycle_n \geq 1$ **then**
  if ($MR_n == 1$ AND $p21_n == 0$ AND $p27_n == 0$) OR $ID2_n == 1$ **then**
    $cycle_n \leftarrow cycle_n$

else
$cycle_n \leftarrow cycle_n - \frac{T_{step}}{T_{G1}}$
end if
else
$cycle_n \leftarrow cycle_n$
end if

DNA replication is the main activity in S phase, requiring cyclin A/CDK2. As discussed above, p21 and p27 can both inhibit CDK2 effectively. Therefore, progression through the S phase is only possible when both species are inactive. During replication, errors may occur randomly or due to hypoxia, which makes such errors more likely [11]. At the end of S, cyclin A/CDC2 facilitates entry into the G2 phase ($2 \leq cycle_n < 3$). However, the CDKIs do not inhibit this complex, so this transition occurs by default.

The agent's progression through S is represented by the following conditional statements, which are implemented when $1 \leq cycle_n < 2$.

if $p21_n == 0$ AND $p27_n == 0$ **then**
$cycle_n \leftarrow cycle_n + \frac{T_{step}}{T_S}$
if $DNA_{unrep,n} == 0$ AND $P_{unrep} \times T_{step} > rand$ **then**
$DNA_{unrep,n} \leftarrow 1$
else
if $DNA_{unrep,n} == 0$ AND $P_{unrep,h} \times T_{step} > rand$ AND $hypoxia_n == 1$ **then**
$DNA_{unrep,n} \leftarrow 1$
else
$DNA_{unrep,n} \leftarrow DNA_{unrep,n}$
end if
end if
else
$cycle_n \leftarrow cycle_n$
end if

In the G2 phase, cyclin A/CDC2 is active. The agent progresses through this phase by default, but there is a cell cycle checkpoint (restriction point) at the end, when cyclin B synthesis begins. Cyclin B/CDC2 is necessary for entry into the M phase. Phosphorylation of CDC2 Thr160 is in turn necessary for cyclin B/CDC2 activation. Opposing this activating mechanism, phosphorylation of CDC2 Thr14 and Tyr15 keeps the complex in an inactive state. Countering this inactivating mechanism, CDC25C dephosphorylates Thr14 and Tyr15. In this mechanism, CDC25B may be involved in dephosphorylating and activating cyclin B/CDC2 to initiate a positive feedback loop wherein CDC25C is activated by CDC2. However, the initial trigger of CDC25C activation is unclear.

The agent's progression through G2 is represented by the following conditional statements, which are implemented when $2 \leq cycle_n < 3$.

$cycle_n \leftarrow cycle_n + \frac{T_{step}}{T_{G2}}$
if $cycle_n \geq 3$ AND $CDC25C_n == 0$ **then**
$cycle_n \leftarrow cycle_n - \frac{T_{step}}{T_{G2}}$
else
$cycle_n \leftarrow cycle_n$
end if

In the M phase ($3 \leq cycle_n < 4$), the agent progresses by default. Therefore, when $3 \leq cycle_n < 4$, the following operation is implemented in the *Cycle* function.

if $3 \leq cycle_n < 4$ **then**
$cycle_n \leftarrow cycle_n + \frac{T_{step}}{T_M}$
else
$cycle_n \leftarrow cycle_n$

end if

At the end of the M phase ($cycle_n == 4$), it divides and both daughter cells return to G0. This transition between two successive cycles is represented by the next transition function: *Divide*.

B.3 Divide

The *Divide* function only applies to a living cell ($apop_n$ and $necro_n$ are both zero).

First, we consider the process of telomere repair, which may occur when $N_{telo,n} < N_{telo,max}$. There are two mutually exclusive repair mechanisms: telomerase ($telo_n$) and ALT (ALT_n). Chemotherapy (fifth element of $chemo_{effects}$) affects the first mechanism. The process is represented by the following conditional statements, which are implemented when $N_{telo,n} < N_{telo,max}$.

if $telo_n == 1$ AND $P_{telo,r} \times T_{step} > rand$ **then**
  $dummy \leftarrow 1$
  if $\exists i : chemo_{start,i} <= t <= chemo_{end,i}$ AND $chemo_{effects,5} > rand$ **then**
    $dummy \leftarrow 0$
  else
    $dummy \leftarrow 1$
  end if
  if $dummy == 1$ **then**
    $N_{telo,n} \leftarrow N_{telo,n} + 1$
  else
    $N_{telo,n} \leftarrow N_{telo,n}$
  end if
else
  if $ALT_n == 1$ AND $P_{telo,r} \times T_{step} > rand$ **then**
    $N_{telo,n} \leftarrow N_{telo,n} + 1$
  else
    $N_{telo,n} \leftarrow N_{telo,n}$
  end if
end if

Second, independently of telomere repair, the *Divide* function describes cell division. This event comprises a reduction in $telo_n$, entry into G0, and a signal for cell division. It is represented by the following conditional statements.

if $cycle_n \geq 4$ **then**
  $cycle_n \leftarrow 0$
  if $N_{telo,n} > 0$ **then**
    $telo_n \leftarrow telo_n - 1$
  else
    $telo_n \leftarrow telo_n$
  end if
  $signal_{divide,n} \leftarrow 1$
else
  $signal_{divide,n} \leftarrow 0$
end if

When $signal_{divide,n}$ is one, a copy of the agent is created. The co-ordinates of both daughter cell agents are updated as follows. After generating three random numbers ($dummy_x$, $dummy_y$, and $dummy_z$) from the uniform distribution between -1 and 1, scaling them (such as $dummy_{x,s} = \frac{dummy_x}{\sqrt{dummy_x^2+dummy_y^2+dummy_z^2}}$), they are used to perturb a daughter cell's coordinates (such as $x_n \leftarrow x_n + L_{cell} \times dummy_{x,s}$).

B.4 Remove

This function only applies to a dead agent ($apop_n$ or $necro_n$ is one). It stochastically removes the dead agent from the system to mimic the immune system's ability to engulf dead cells.

if $P_{lysis} \times T_{step} > rand$ **then**
  $signal_{remove,n} \leftarrow 1$
else
  $signal_{remove,n} \leftarrow 0$
end if

C Schwann cell agents

Each Schwann cell agent's state is defined by three types of attributes, which are recorded in two vectors (physical and cellular) and a Boolean variable (ATP availability). The agent is denoted by the index *sc*.

1. Physical vector: $physical_{sc} = (x_{sc}, y_{sc}, z_{sc}, F_{x,sc}, F_{y,sc}, F_{z,sc}, \delta_{sc}^{overall}, N_{nghbr,sc}, mobile_{sc})$. These are the agent's spatial coordinates in microns, the net forces acting on it in the three spatial dimensions (N), its overlap with the other agents in the system (only positive and in microns), the number of agents in its neighbourhood (including itself), and a Boolean variable indicating whether it is mobile and can generate a locomotive force.

2. Cellular vector: $cellular_{sc} = (cycle_{sc}, apop_{sc}, signal_{apop,sc}, necro_{sc}, signal_{necro,sc}, signal_{necro,c}, N_{telo,sc}, hypoxia_{sc}, nutrient_{sc}, DNA_{damage,sc}, DNA_{unrep,sc})$. $cycle_{sc}$ is a continuous variable labelling the agent's position in the cell cycle (zero to four; zero means G0, one means G1/S, two means S/G2, three means G2/M, and four signals division [3]). $apop_{sc}$ is a Boolean variable indicating whether the agent is apoptotic. $signal_{apop,sc}$ is a discrete variable representing the number of apoptotic signals the agent has. The time from the initiation of apoptosis to its completion is as short as two or three hours [4]. $necro_{sc}$ is a Boolean variable indicating whether the agent is necrotic. $signal_{necro,sc}$ is a discrete variable representing the number of necrotic signals the agent has. $signal_{necro,c}$ is a discrete variable representing the number of necrotic signals it needs to turn necrotic (necrosis occurs over prolonged periods from a few hours to approximately one week [5]). $N_{telo,sc}$ is a discrete variable representing its telomere lengths abstractly (a normal human foetal cell can divide between 40 and 60 times before entering a senescence phase due to shortening telomeres [6]). $hypoxia_{sc}$ is a Boolean variable indicating whether the agent is hypoxic, while $nutrient_{sc}$ is another Boolean variable indicating whether it has access to enough nutrients. $DNA_{damage,sc}$ is a Boolean variable indicating whether the agent has damaged DNA and $DNA_{unrep,sc}$ is a Boolean variable indicating whether it has unreplicated DNA.

3. ATP_{sc} is a Boolean variable indicating whether the agent has sufficient ATP.

During a time step, the following transition functions are used to update the state vectors and ATP_{sc}: *Sense*, *Cycle*, *Divide*, and *Remove*. The main difference between this set of functions and the set associated with a neuroblastoma cell agent is that this set does not consider any intracellular gene products, which are not present in a Schwann cell agent. Another difference is that a Schwann cell agent cannot differentiate. Finally, without telomerase or ALT, it cannot repair its telomeres.

C.1 Sense

This function represents the tumour's microenvironment's influence on the Schwann cell represented by an agent. It changes the agent's intracellular conditions to influence its phenotypic behaviours (subsequent transition functions). Unless otherwise stated, the source of this subsection is a book chapter [3]. These are the specific updates encapsulated within this function.

1. Responses to stressors: hypoxia and lack of glucose.

2. Changes to DNA.

3. Responses to necrotic signals.

4. Responses to apoptotic signals.

5. Apoptosis and necrosis.

There are two major stressors. First, as in [5], hypoxia-induced necrosis is a saturable process in our model and the probability of hypoxia is $1 - \frac{C^s_{O2} \times C_{O2}}{C^s_{O2} \times C_{O2} + C^{50}_{O2}}$. Second, the cell agent requires other nutrients, such as glucose. Oxygen availability is considered a sufficient proxy for resource availability [5], partly because oxygen and other resources are collectively distributed by the vasculature, so their concentration profiles should be highly correlated.

if $1 - \frac{C^s_{O2} \times C_{O2}}{C^s_{O2} \times C_{O2} + C^{50}_{O2}} > rand$ **then**
  $hypoxia_{sc} \leftarrow 1$
else
  $hypoxia_{sc} \leftarrow 0$
end if

if $1 - \frac{C^s_{O2} \times C_{O2}}{C^s_{O2} \times C_{O2} + C^{50}_{O2}} > rand$ **then**
  $nutrient_{sc} \leftarrow 0$
else
  $nutrient_{sc} \leftarrow 1$
end if

A Schwann cell agent's DNA gets damaged due to having shortening telomeres, hypoxia, and chemotherapy. They are three independent mechanisms.

1. A normal human foetal cell can divide between 40 and 60 times before reaching the Hayflick limit and entering a senescence phase due to shortening telomeres [6]. After 40 divisions, it will become increasingly susceptible to shortening telomeres. In the absence of any telomere repair mechanisms (telomerase or ALT pathway), the Schwann cell agent's probability of having damaged DNA will become 100 % after 60 divisions. In our model, a cell agent with damaged DNA is susceptible to apoptosis, but it is acknowledged that after reaching the Hayflick limit, a real cell actually becomes senescent: it stops cycling, but does not always die [9]. However, like apoptotic cells, senescent cells are engulfed by the immune system [10]. For the sake of simplicity, senescent cell agents are considered to have damaged DNA (apoptotic).
2. The chance that the agent's DNA is damaged is increased during hypoxia [11].
3. Chemotherapy damages the agent's DNA in the S phase of the cell cycle. As the Schwann cell agent does not have any intracellular proteins, the relevant inhibitory effects of chemotherapy are implemented directly. The six elements of $chemo_{effects}$ are averaged to produce an overall effect on the agent's DNA.

Opposing these DNA-damaging mechanisms, the agent can repair its damaged DNA naturally when it is not under the influence of chemotherapy.

These changes to the agent's DNA are represented by the following conditional statements.

if $\exists i : chemo_{start,i} <= t <= chemo_{end,i}$ AND $mean(chemo_{effects}) > rand$ **then**
  $dummy \leftarrow 1$
else
  $dummy \leftarrow 0$
end if

if $DNA_{damage,sc} == 0$ **then**
  if $(1 - \frac{N_{telo,sc}}{N_{telo,c}}) \times T_{step} > rand$ **then**
    $DNA_{damage,sc} \leftarrow 1$
  else
    if $hypoxia_{sc} == 1$ AND $P_{DNA,h} \times T_{step} > rand$ **then**
      $DNA_{damage,sc} \leftarrow 1$
    else

if $dummy == 1$ AND $1 < cycle_{sc} < 2$ AND $P_{DNA,c} \times T_{step} > rand$ **then**
$DNA_{damage,sc} \leftarrow 1$
else
$DNA_{damage,sc} \leftarrow DNA_{damage,sc}$
end if
end if
end if
else
if $dummy == 0$ AND $P_{DNA,r1} \times T_{step} > rand$ **then**
$DNA_{damage,sc} \leftarrow 0$
else
$DNA_{damage,sc} \leftarrow DNA_{damage,sc}$
end if
end if

The *Sense* function also gives the agent a chance to repair any unreplicated DNA when it is not under the influence of chemotherapy. This operation is represented by the following conditional statements.

if $\exists i : chemo_{start,i} <= t <= chemo_{end,i}$ AND $mean(chemo_{effects}) > rand$ **then**
$dummy \leftarrow 1$
else
$dummy \leftarrow 0$
end if

if $DNA_{unrep,sc} == 1$ **then**
if $dummy == 0$ AND $P_{DNA,r2} \times T_{step} > rand$ **then**
$DNA_{unrep,sc} \leftarrow 0$
else
$DNA_{unrep,sc} \leftarrow DNA_{unrep,sc}$
end if
else
$DNA_{unrep,sc} \leftarrow DNA_{unrep,sc}$
end if

Two potentially additive sources of necrotic signals are considered: necrotic cell agents in the living agent's 3D von Neumann neighbourhood and glycolysis caused by deficiency in nutrients. They are additive in the sense that the two triggers are not mutually exclusive and may co-occur in one time step.

First, necrotic cells release their contents in an uncontrolled manner, including microbial substances that can damage living cells to cause inflammation [12].

The second trigger for necrosis depends on the availability of ATP and how it is generated. When there is enough glucose but not enough oxygen, ATP is generated by glycolysis, which releases lactic acid and is approximately less effective than oxidative phosphorylation by a factor of 15 [13].

After considering both sources, if the cell agent does not have at least one new necrotic signal, it may lose some of its necrotic signals (recovery).

These operations are represented by the following conditional statements.

$dummy \leftarrow 0$
$stress \leftarrow 0$

while $dummy < \sum_{3DVNN}(N^n_{i,j,k} + S^n_{i,j,k})$ **do**
if $P^{is}_{necro} \times T_{step} > rand$ **then**
increase $signal_{necro,sc}$ by T_{step}
$stress \leftarrow 1$

end if
increase $dummy$ by one
end while

if $nutrient_{sc} == 1$ **then**
if $hypoxia_{sc} == 0$ **then**
$ATP_{sc} \leftarrow 1$
if $signal_{necro,sc} > 0$ AND $stress == 0$ AND $P_{necro,r} \times T_{step} > rand$ **then**
reduce $signal_{necro,sc}$ by T_{step}
end if
else
if $R_{glycolysis} > rand$ **then**
$ATP_{sc} \leftarrow 1$
increase $signal_{necro,sc}$ by T_{step}
else
$ATP_{sc} \leftarrow 0$
increase $signal_{necro,sc}$ by $2 \times T_{step}$
end if
end if
else
$ATP_{sc} \leftarrow 0$
increase $signal_{necro,sc}$ by T_{step}
end if

The agent's number of apoptotic signals will be updated next. There are three mutually exclusive mechanisms for the Schwann cell agent to acquire apoptotic signals. First, hypoxia ($hypoxia_{sc}$ is one) activates CAS to induce apoptosis when the agent has enough ATP (ATP_{sc} is one). Second, damaged DNA ($DNA_{damage,sc}$ is one) activates CAS when the agent has enough ATP and when it is not under the influence of chemotherapy, which disrupts the pathways linking damaged DNA to CAS. Since the agent represents a Schwann cell and does not have an attribute representing CAS, $hypoxia_{sc}$ and $DNA_{damage,sc}$ are linked to apoptosis directly. There is a third CAS-independent mechanism because, even without CAS, a cell with damaged DNA just cannot survive and the second mechanism is inactive under the influence of chemotherapy. If the agent does not acquire an apoptotic signal through these mechanisms and it still has a non-zero number of signals, it may lose some of them. The following conditional statements represent these mechanisms in our model.

if $\exists i : chemo_{start,i} <= t <= chemo_{end,i}$ AND $mean(chemo_{effects}) > rand$ **then**
$dummy \leftarrow 1$
else
$dummy \leftarrow 0$
end if

$stress \leftarrow 0$

if $hypoxia_{sc} == 1$ AND $ATP_{sc} == 1$ **then**
$signal_{apop,sc} \leftarrow signal_{apop,sc} + T_{step}$
$stress \leftarrow 1$
else
if $DNA_{damage,sc} == 1$ AND $ATP_{sc} == 1$ AND $dummy == 0$ **then**
$signal_{apop,sc} \leftarrow signal_{apop,sc} + T_{step}$
$stress \leftarrow 1$
else
if $DNA_{damage,sc} == 1$ AND $ATP_{sc} == 1$ AND $P_{apop} \times T_{step} > rand$ **then**
$signal_{apop,sc} \leftarrow signal_{apop,sc} + T_{step}$
$stress \leftarrow 1$
else
$signal_{apop,sc} \leftarrow signal_{apop,sc}$
$stress \leftarrow stress$

end if
end if
end if

if $stress == 0$ AND $signal_{apop,sc} > 0$ AND $P_{apop,r} \times T_{step} > rand$ **then**
$signal_{apop,sc} \leftarrow signal_{apop,sc} - T_{step}$
else
$signal_{apop,sc} \leftarrow signal_{apop,sc}$
end if

Finally, the agent's apoptotic status and necrotic status are upated. Apoptotic cells in any system die and disappear relatively quickly, as quickly as two or three hours after initiation [4]. As for necrosis, hypoxia can cause necrosis over a prolonged period ranging from a few hours to approximately one week [5]. In addition, an apoptotic cell may undergo secondary necrosis [32]. Intuitively, an apoptotic or necrotic cell is immobile and has no ATP. The following conditional statements are used to implement these updates.

if $signal_{apop,sc} >= T_{apop}$ **then**
$mobile_{sc} \leftarrow 0$
$ATP_{sc} \leftarrow 0$
$apop_{sc} \leftarrow 1$
else
if $signal_{necro,sc} >= signal_{necro,c}$ **then**
$mobile_{sc} \leftarrow 0$
$ATP_{sc} \leftarrow 0$
$necro_{sc} \leftarrow 1$
else
if $apop_{sc} == 1$ AND $necro_{sc} == 0$ AND $P_{necro,2} \times T_{step} > rand$ **then**
$apop_{sc} \leftarrow 0$
$necro_{sc} \leftarrow 1$
else
$apop_{sc} \leftarrow apop_{sc}$
$necro_{sc} \leftarrow necro_{sc}$
end if
end if
end if

C.2 Cycle

This function models a Schwann cell agent's progress through the cell cycle. Unless otherwise stated, the source of this subsection is a book chapter [3].

The agent can only make progress in the cell cycle if all of the following conditions are met.

1. The agent must be proliferative and this condition can be met in three ways: juxtacrine signals from the neuroblastoma cell agents in its neighbourhood [30], paracrine signals from the neuroblastoma cell agents in the whole continuous automaton [30], and its intrinsic tendency to proliferate.
2. It must not be inhibited by its immediate neighbours (contact inhibition) [33].
3. It must have enough ATP to meet the energy requirement of cell cycling [33].
4. By intuition, only a living cell can cycle, so it must be neither apoptotic nor necrotic.

Unlike a neuroblastoma cell agent, which is encouraged to differentiate and undergo apoptosis by its neighbouring Schwann cell agents (*Sense* function), non-mechanical cell-cell interactions affect the cycling ability of Schwann cell agents directly (*Cycle* function) [30]. The key metric for juxatacrine signalling is the ratio of the number of living neuroblastoma cell agents to the number of living cell agents in this agent's 3D von Neumann neighbourhood: $ratio_{3DVNN} = \frac{\sum_{3DVNN} N^l_{i,j,k}}{\sum_{3DVNN} N^l_{i,j,k} + \sum_{3DVNN} S^l_{i,j,k}}$. The key metric for paracrine signalling is the ratio of the number of living neuroblastoma cell agents to the number of

living cell agents in the entire continuous automaton: $ratio_{CA} = \frac{\sum_{CA} N^l_{i,j,k}}{\sum_{CA} N^l_{i,j,k} + \sum_{CA} S^l_{i,j,k}}$.

The conditional statements representing the above conditions are implemented at the start of the *Cycle* function.

$dummy \leftarrow 0$

if $R^{jux}_{pro,sc} \times ratio_{3DVNN} \times T_{step} > rand$ **then**
  $dummy \leftarrow 1$
else
  if $R^{para}_{pro,sc} \times ratio_{CA} \times T_{step} > rand$ **then**
    $dummy \leftarrow 1$
  else
    $dummy \leftarrow 0$
  end if
end if

if $(dummy == 1$ OR $P_{cycle,sc} > rand)$ AND $N_{nghbr,sc} <= N_{nghbr,max}$ AND $ATP_{sc} == 1$ AND $apop_{sc} == 0$ AND $necro_{sc} == 0$ **then**
  do nothing
else
  exit *Cycle* function
end if

Just like a neuroblastoma cell, a Schwann cell has a cell cycle comprising distinct phases: G0 (quiescent or resting), G1, S, G2 and M. The non-quiescent phases last 12 (T_{G1}), six (T_S), four (T_{G2}), and two (T_M) hours respectively. Its radius increases from $\frac{L_{cell}}{2}$ to L_{cell} as it progresses through G1 and G2. The increase in its radius is split into 16 parts corresponding to the 12 and four hours constituting G1 and G2 respectively. For example, during G1, its radius is $\frac{L_{cell}}{2} + \frac{L_{cell}}{2} \times \frac{12 \times cycle_{sc}}{16}$.

The agent enters the G0 phase ($cycle_{sc}$ is zero) immediately after dividing. As discussed, damaged DNA (caused by chemotherapy and other factors) and hypoxia both switch on p21 and p27, which inhibit various cyclin/CDK complexes. Since the Schwann cell agent does not have any intracellular proteins, these effects are implemented directly by linking $DNA_{damage,sc}$ and $hypoxia_{sc}$ to $cycle_{sc}$. When $cycle_{sc}$, $DNA_{damage,sc}$, and $hypoxia_{sc}$ are all zero, increment $cycle_{sc}$ by $\frac{T_{step}}{T_{G1}}$.

After leaving G0 and during the G1 phase ($0 < cycle_{sc} < 1$), cell cycle progression follows the same logic. When $0 < cycle_{sc} < 1$, $DNA_{damage,sc}$ is zero, and $hypoxia_{sc}$ is zero, increment $cycle_{sc}$ by $\frac{T_{step}}{T_{G1}}$. The G1/S checkpoint follows the same logic.

The S phase ($1 \leq cycle_{sc} < 2$) follows the same logic as G1 except that the agent's DNA may become unreplicated. The following conditional statements are implemented when $1 \leq cycle_{sc} < 2$.

if $DNA_{damage,sc} == 0$ And $hypoxia_{sc} == 0$ **then**
  $cycle_{sc} \leftarrow cycle_{sc} + \frac{T_{step}}{T_S}$
  if $DNA_{unrep,sc} == 0$ AND $P_{unrep} \times T_{step} > rand$ **then**
    $DNA_{unrep,sc} \leftarrow 1$
  else
    if $DNA_{unrep,sc} == 0$ AND $P_{unrep,h} \times T_{step} > rand$ **then**
      $DNA_{unrep,sc} \leftarrow 1$
    else
      $DNA_{unrep,sc} \leftarrow DNA_{unrep,sc}$
    end if
  end if
else
  $cycle_{sc} \leftarrow cycle_{sc}$
end if

At the end of S, the agent enters the G2 phase ($2 \leq cycle_{sc} < 3$) by default. During G2, it progresses in the cell cycle by default. When $2 \leq cycle_{sc} < 3$, increment $cycle_{sc}$ by $\frac{T_{step}}{T_{G2}}$. After this operation, implement another conditional statement, which represents the G2/M checkpoint. If $cycle_{sc} \geq 3$ and $DNA_{damage,sc}$ or $DNA_{unrep,sc}$ is one, reduce $cycle_{sc}$ by $\frac{T_{step}}{T_{G2}}$. As discussed, both damaged and unreplicated DNA switch off CDC25C to arrest the cell cycle at this checkpoint. Since the Schwann cell agent does not have any intracellular proteins, these effects are implemented directly by linking $DNA_{damage,sc}$ and $DNA_{unrep,sc}$ to $cycle_{sc}$.

During the M phase ($3 \leq cycle_{sc} < 4$), a cell progresses by default. Therefore, when $3 \leq cycle_{sc} < 4$, increment $cycle_{sc}$ by $\frac{T_{step}}{T_M}$. At the end of the M phase ($cycle_{sc}$ is four), it divides and both daughter cells return to G0. This transition between successive cycles is represented by the following *Divide* function.

C.3 Divide

The *Divide* function makes three changes to a living Schwann cell agent ($apop_{sc}$ and $necro_{sc}$ are both zero) when $cycle_{sc} \geq 4$. First, it sets $cycle_{sc}$ back to zero. Second, if $N_{telo,sc} > 0$, reduce $N_{telo,sc}$ by one. Third, the function returns a signal for cell division by setting $signal_{divide,sc}$ to one. If $cycle_{sc} < 4$, set $signal_{divide,sc}$ to zero.

If $signal_{divide,sc}$ is one, three random numbers ($dummy_x$, $dummy_y$, and $dummy_z$) are generated from the uniform distribution between -1 and 1, scaled (such as $dummy_{x,s} = \frac{dummy_x}{\sqrt{dummy_x^2 + dummy_y^2 + dummy_z^2}}$), and used to perturb each daughter cell's coordinates (such as $x_{sc} \leftarrow x_{sc} + L_{cell} \times dummy_{x,s}$).

C.4 Remove

This function only applies to a dead agent ($apop_{sc}$ or $necro_{sc}$ is one), stochastically removing it from the system to mimic the immune system's ability to engulf dead cells.

if $P_{lysis} \times T_{step} > rand$ **then**
  $signal_{remove,sc} \leftarrow 1$
else
  $signal_{remove,sc} \leftarrow 0$
end if

D Mechanical model

As explained in the main article, the mechanical model comprises a linear force law and an equation of motion. It is an off-lattice model allowing for continuous changes in x_n, y_n, z_n, x_s, y_s, and z_s. The origin is the spatial domain's centre at the start of a simulation. The force law relates the overlap ($\delta_{1,2}$) between any two cells (displacement vectors $\vec{r_1}$ and $\vec{r_2}$) in the simulation to the repulsive force acting between them. The two equations constituting this law are reproduced here as a reminder.

$$\delta_{1,2} = R_1 + R_2 - \|\vec{r_1} - \vec{r_2}\|. \quad (A)$$

$$F_{1,2} = k_1 \delta_{1,2}. \quad (B)$$

What is not stated in the main article is that if an agent is paired up with itself or if $\delta_{1,2} < L_{overlap}$, $\delta_{1,2}$ is set to zero. Furthermore, if $\|\vec{r_1} - \vec{r_2}\| < L_{nghbr}$, $N_{nghbr,n}$ or $N_{nghbr,sc}$ is incremented by one. As explained in the article, the force vectors linking the agent to its peers are summed to produce a net force vector, which is multiplied by k_2 if $N_{nghbr,n} > N_{nghbr,max}$ or $N_{nghbr,sc} > N_{nghbr,max}$ and the agent is mobile ($mobile_n$ or $mobile_{sc}$ is one). After this operation, the net force vector, (F^x, F^y, F^z), will become ($F_{x,n}$, $F_{y,n}$, $F_{z,n}$) or ($F_{x,sc}$, $F_{y,sc}$, $F_{z,sc}$).

The following equation of motion describes how the agent moves in response to this net force in the x direction:

$$F^x = \mu(1+M)\frac{dx}{dt}. \tag{C}$$

This equation and its counterparts in the other two spatial dimensions are implemented computationally by Euler's method. For example, the x-coordinate (x_n or x_{sc}) is incremented by $\frac{F_{x,n}\Delta t}{\mu(1+M)}$ or $\frac{F_{x,sc}\Delta t}{\mu(1+M)}$, where Δt is the size of each update in seconds. If this update causes the agent to go beyond one of the continuous automaton's six boundaries (determined by k_3), it will be pushed back by k_4 times its distance from the boundary.

By cycling between updating the force law and implementing the equations of motion, a mechanical equilibrium can be identified for the population of agents in the simulation. After updating every agent's spatial coordinates, four independent conditions for termination (mechanical equilibrium) are checked at the end of an iteration. The first condition is met when there are fewer than two agents in the simulation. The second condition entails that $N_{nghbr,n} \leq N_{nghbr,max}$ and $N_{nghbr,sc} \leq N_{nghbr,max}$ $\forall(n, sc)$. The third condition activates when the maximum total overlap experienced by an agent belonging to the population is less than 15 % of $\frac{L_{cell}}{2}$. The fourth condition is an upper limit on the number of iterations (force resolution steps): $\frac{3600\times T_{step}}{\Delta t}$.

E Linking functions

There are two functions that pass information between the three parts making up the hybrid model. They represent the vasculature and update the extracellular matrix/oxygen concentrations respectively.

E.1 Vasculature

This function provides a simplified representation of a tumour's microvasculature, including its growth and how it supplies oxygen to support the living cells in the tumour.

It is applied at the beginning of each simulation to initialise two variables. First, the rate at which oxygen is supplied to the whole continuous automaton (R_{O2}) is set to the initial oxygen consumption rate therein: $-R^0_{O2} \times \frac{\sum_{CA}(N^l_{i,j,k}+S^l_{i,j,k})}{N_{vox}\times L^3_{voxel}\times C^s_{O2}}$, where N_{vox} is the total number of voxels in the continuous automaton. In addition, the integral number of angiogenic signals in the system (N_{ang}) is set to zero.

It is known that neuroblastoma cells stimulate angiogenesis by releasing VEGF [39] and Schwann cells inhibit angiogenesis [40]. Therefore, during each subsequent time interval, the integral number of VEGF-producing neuroblastoma cell agents in the entire continuous automaton (N_{vf}) is compared to the number of living Schwann cell agents therein ($\sum_{CA} S^l_{i,j,k}$). If $N_{vf} > \sum_{CA} S^l_{i,j,k}$, N_{ang} is increased by T_{step}. Recall that the timescale is T^c_{ang}, which is measured in hours. After updating N_{ang}, the function implements the following conditional statements.

if $N_{ang} == T^c_{ang}$ **then**
    Update $N^l_{i,j,k}$ and $S^l_{i,j,k}$.
    Update N_{vox}.
    $dummy \leftarrow -R^0_{O2} \times \frac{\sum_{CA}(N^l_{i,j,k}+S^l_{i,j,k})}{N_{vox}\times L^3_{voxel}\times C^s_{O2}}$
    if $dummy > R_{O2}$ **then**
        $R_{O2} \leftarrow dummy$
    else
        $R_{O2} \leftarrow R_{O2}$
    end if
    $N_{ang} \leftarrow 0$
else
    $R_{O2} \leftarrow R_{O2}$
    $N_{ang} \leftarrow N_{ang}$
end if

E.2 Continuous automaton update

The second linking function updates the dimensionless and spatially homogeneous oxygen level (C_{O2}) and the fraction of volume occupied by extracellular matrix in each voxel ($M_{i,j,k}$ in $voxstate_{i,j,k}$). The first update is overlooked when $static_{O2}$ is one.

if $static_{O2} == 0$ **then**
  $C_{O2} \leftarrow C_{O2} + R^0_{O2} \times \frac{\sum_{CA}(N^l_{i,j,k}+S^l_{i,j,k})}{N_{vox}\times L^3_{voxel}\times C^s_{O2}} \times T_{step}$
  $C_{O2} \leftarrow C_{O2} + R_{O2}$
  if $C_{O2} > 1$ **then**
    $C_{O2} \leftarrow 1$
  else
    if $C_{O2} < 0$ **then**
      $C_{O2} \leftarrow 0$
    else
      $C_{O2} \leftarrow C_{O2}$
    end if
  end if
else
  $C_{O2} \leftarrow C_{O2}$
end if

The second update assumes the extracellular matrix to be immobile and its degradation to be negligible on this time scale. For each voxel in the continuous automaton, the following is implemented: $M_{i,j,k} \leftarrow M_{i,j,k} + \frac{S^{lm}_{i,j,k}\times R_{collagen}\times T_{step}}{L^3_{voxel}}$.

References

[1] Carreau A, Hafny-Rahbi BE, Matejuk A, Grillon C, Kieda C. Why is the partial oxygen pressure of human tissues a crucial parameter? Small molecules and hypoxia. Journal of cellular and molecular medicine. 2011;15(6):1239-53.

[2] Neumann A, Alexander B, Neumann F. Evolutionary image transition and painting using random walks. Evolutionary computation. 2020;28(4):643-75.

[3] Harper JV, Brooks G. The mammalian cell cycle: an overview. Cell Cycle Control: Mechanisms and Protocols. 2005:113-53.

[4] Elmore S. Apoptosis: a review of programmed cell death. Toxicologic pathology. 2007;35(4):495-516.

[5] Warren DR, Partridge M. The role of necrosis, acute hypoxia and chronic hypoxia in 18F-FMISO PET image contrast: a computational modelling study. Physics in Medicine & Biology. 2016;61(24):8596.

[6] Hayflick L, Moorhead PS. The serial cultivation of human diploid cell strains. Experimental cell research. 1961;25(3):585-621.

[7] Smith ML, Seo YR. p53 regulation of DNA excision repair pathways. Mutagenesis. 2002;17(2):149-56.

[8] Zaika E, Wei J, Yin D, Andl C, Moll U, El-Rifai W, et al. p73 protein regulates DNA damage repair. The FASEB journal. 2011;25(12):4406-14.

[9] Liu J, Wang L, Wang Z, Liu JP. Roles of telomere biology in cell senescence, replicative and chronological ageing. Cells. 2019;8(1):54.

[10] Song P, An J, Zou MH. Immune clearance of senescent cells to combat ageing and chronic diseases. Cells. 2020;9(3):671.

[11] Muz B, de la Puente P, Azab F, Azab AK. The role of hypoxia in cancer progression, angiogenesis, metastasis, and resistance to therapy. Hypoxia. 2015;3:83.

[12] Rock KL, Kono H. The inflammatory response to cell death. Annu Rev Pathol Mech Dis. 2008;3:99-126.

[13] du Plessis SS, Agarwal A, Mohanty G, Van der Linde M. Oxidative phosphorylation versus glycolysis: what fuel do spermatozoa use? Asian journal of andrology. 2015;17(2):230.

[14] Huang M, Weiss WA. Neuroblastoma and MYCN. Cold Spring Harbor perspectives in medicine. 2013;3(10):a014415.

[15] Greijer A, Van der Wall E. The role of hypoxia inducible factor 1 (HIF-1) in hypoxia induced apoptosis. Journal of clinical pathology. 2004;57(10):1009-14.

[16] Larsen M, Høg A, Lund EL, Kristjansen PE. Interactions between HIF-1 and Jab1: Balancing apoptosis and adaptation: Outline of a working hypothesis. Oxygen Transport to Tissue XXVI. 2005:203-11.

[17] Amelio I, Inoue S, Markert EK, Levine AJ, Knight RA, Mak TW, et al. TAp73 opposes tumor angiogenesis by promoting hypoxia-inducible factor 1α degradation. Proceedings of the National Academy of Sciences. 2015;112(1):226-31.

[18] Wang Y, Wang X, Flores ER, Yu J, Chang S. Dysfunctional telomeres induce p53-dependent and independent apoptosis to compromise cellular proliferation and inhibit tumor formation. Aging cell. 2016;15(4):646-60.

[19] Chen D, Li M, Luo J, Gu W. Direct interactions between HIF-1α and Mdm2 modulate p53 function. Journal of Biological Chemistry. 2003;278(16):13595-8.

[20] Tang XX, Zhao H, Kung B, Kim DY, Hicks SL, Cohn SL, et al. The MYCN enigma: significance of MYCN expression in neuroblastoma. Cancer research. 2006;66(5):2826-33.

[21] Chen L, Iraci N, Gherardi S, Gamble LD, Wood KM, Perini G, et al. p53 is a direct transcriptional target of MYCN in neuroblastoma. Cancer research. 2010;70(4):1377-88.

[22] Dulloo I, Hooi PB, Sabapathy K. Hypoxia-induced DNp73 stabilization regulates Vegf-A expression and tumor angiogenesis similar to TAp73. Cell cycle. 2015;14(22):3533-9.

[23] Goda N, Ryan HE, Khadivi B, McNulty W, Rickert RC, Johnson RS. Hypoxia-inducible factor 1α is essential for cell cycle arrest during hypoxia. Molecular and cellular biology. 2003;23(1):359-69.

[24] Hubbi ME, Semenza GL. Regulation of cell proliferation by hypoxia-inducible factors. American Journal of Physiology-Cell Physiology. 2015;309(12):C775-82.

[25] Michieli P, Chedid M, Lin D, Pierce JH, Mercer WE, Givol D. Induction of WAF1/CIP1 by a p53-independent pathway. Cancer research. 1994;54(13):3391-5.

[26] Matsui TA, Murata H, Sowa Y, Sakabe T, Koto K, Horie N, et al. A novel MEK1/2 inhibitor induces G1/S cell cycle arrest in human fibrosarcoma cells. Oncology reports. 2010;24(2):329-33.

[27] Eckerle I, Muth D, Batzler J, Henrich KO, Lutz W, Fischer M, et al. Regulation of BIRC5 and its isoform BIRC5-2B in neuroblastoma. Cancer letters. 2009;285(1):99-107.

[28] Ruiz-Pérez MV, Henley AB, Arsenian-Henriksson M. The MYCN protein in health and disease. Genes. 2017;8(4):113.

[29] Yoon MK, Ha JH, Lee MS, Chi SW. Structure and apoptotic function of p73. BMB reports. 2015;48(2):81.

[30] Ambros IM, Attarbaschi A, Rumpler S, Luegmayr A, Turkof E, Gadner H, et al. Neuroblastoma cells provoke Schwann cell proliferation in vitro. Medical and Pediatric Oncology: The Official Journal of SIOP—International Society of Pediatric Oncology (Societé Internationale d'Oncologie Pédiatrique). 2001;36(1):163-8.

[31] Cooper GM. The cell : a molecular approach / Geoffrey M. Cooper. Second edition. ed. Washington, D.C: ASM Press; 2000.

[32] Dunster JL, Byrne HM, King JR. The resolution of inflammation: a mathematical model of neutrophil and macrophage interactions. Bulletin of mathematical biology. 2014;76(8):1953-80.

[33] Jagiella N, Müller B, Müller M, Vignon-Clementel IE, Drasdo D. Inferring growth control mechanisms in growing multi-cellular spheroids of NSCLC cells from spatial-temporal image data. PLoS computational biology. 2016;12(2):e1004412.

[34] Gartel AL, Radhakrishnan SK. Lost in transcription: p21 repression, mechanisms, and consequences. Cancer research. 2005;65(10):3980-5.

[35] Harper JW, Elledge SJ, Keyomarsi K, Dynlacht B, Tsai LH, Zhang P, et al. Inhibition of cyclin-dependent kinases by p21. Molecular biology of the cell. 1995;6(4):387-400.

[36] Ray A, James MK, Larochelle S, Fisher RP, Blain SW. p27Kip1 inhibits cyclin D-cyclin-dependent kinase 4 by two independent modes. Molecular and cellular biology. 2009;29(4):986-99.

[37] Tsytlonok M, Sanabria H, Wang Y, Felekyan S, Hemmen K, Phillips AH, et al. Dynamic anticipation by Cdk2/Cyclin A-bound p27 mediates signal integration in cell cycle regulation. Nature communications. 2019;10(1):1676.

[38] Zetterberg A, Larsson O, Wiman KG. What is the restriction point? Current opinion in cell biology. 1995;7(6):835-42.

[39] Ribatti D, Marimpietri D, Pastorino F, Brignole C, Nico B, Vacca A, et al. Angiogenesis in neuroblastoma. Annals of the New York Academy of Sciences. 2004;1028(1):133-42.

[40] Weiss T, Taschner-Mandl S, Janker L, Bileck A, Rifatbegovic F, Kromp F, et al. Schwann cell plasticity regulates neuroblastic tumor cell differentiation via epidermal growth factor-like protein 8. Nature communications. 2021;12(1):1-19.
