# Supplementary material for: Multicellular model of neuroblastoma proposes unconventional therapy based on multiple roles of p53
Source: PLoS Comput Biol. 2024 Dec 23;20(12):e1012648. doi: 10.1371/journal.pcbi.1012648 (PMC11723635; doi:10.1371/journal.pcbi.1012648)
Supplement: S2 Text — This text explains how we used results from the literature to parameterise the model. (PDF) [file pcbi.1012648.s002.pdf]

S2 Text: Model Parameterisation

Kenneth Y. Wertheim[1,2,3,4], Robert Chisholm[2], Paul Richmond[2], Dawn Walker[1,2]

[1]Insigneo Institute for *in Silico* Medicine, University of Sheffield, Sheffield, UK.
[2]Department of Computer Science, University of Sheffield, Sheffield, UK.
[3]Centre of Excellence for Data Science, Artificial Intelligence, and Modelling, University of Hull, Kingston upon Hull, UK.
[4]School of Computer Science, University of Hull, Kingston upon Hull, UK.

The parametric values and their sources are tabulated in the main article. This text provides more details regarding some of the parameters.

A Macroscopic tumour properties

According to a paper [1], in a tumour with small cells and limited stroma, there are around 1e^{-3} cells µm^{-3}. However, because cells have different sizes and shapes, ρ can be as low as 3.7e^{-5} µm^{-3} in a tumour without extracellular structures. Since L_{cell} is 11 microns, an agent has a diameter of 22 microns at its peak in the cell cycle. Assuming a cubic shape, it means that ρ is $9.39e^{-5}$ µm^{-3}.

B Telomere properties

The maximum telomere length ($N_{telo,max}$) is 60 because a normal human foetal cell population can divide between 40 and 60 times before reaching the Hayflick limit and entering a senescence phase due to shortening telomeres [2]. Every time an agent divides, its $N_{telo,n}$ or $N_{telo,sc}$ is reduced by one. For the same reason, $N_{telo,c}$ is 20.

C Necrosis

The partial pressure of oxygen at which half of the living cells in the spatial domain should be necrotic is 1.2 mmHg, which is converted to C^{50}_{O2} (1.2/2.2779e-4/32 M [3]). The Henry's law constant (2.2779e-4 m^3 mmHg kg^{-1} [4]) for oxygen gas at human body temperature is used for conversion. The molar mass of oxygen is 32 g.

D Intracellular gene products

According to a mechanistic classification scheme [5], assessment of telomere maintenance mechanisms (telomerase and ALT) and a limited set of RAS and p53 pathway genes may be sufficient to accurately estimate patient risk at diagnosis and to guide treatment stratification. As HIF plays a key role in the hypoxic response and p73 shares many functions with p53, they are important too. Therefore, the expression levels of these genes were calibrated. The remaining expression levels are just one.

The activity levels of MYCN and the MAPK/RAS pathway depend on the mutation profile in a neuroblastoma cell, specifically MA_n and ALK_n. By intuition, *MYCN* amplification should alter the activity level of MYCN, but ALK (not the other members in the MAPK/RAS pathway) is also known to regulate MYCN activity positively [6]. By intuition, the activity level of the MAPK/RAS pathway depends on ALK and the other members in the pathway, but *MYCN* amplification is also associated with the pathway's activation [7].

Table A: Activity levels of MYCN given different combinations of mutations in the *MYCN* gene (MA_n) and the genes encoding the MAPK/RAS pathway (ALK_n). E_{MYCN} was obtained by calibration.

	$ALK_n = 0$	$ALK_n = 1$	$ALK_n = 2$
$MA_n = 0$	0.71*0.8*0.94	0.8*0.94	0.71*0.8*0.94
$MA_n = 1$	0.71*0.94	0.94 (E_{MYCN})	0.71*0.94

Table B: Activity levels of the MAPK/RAS pathway given different combinations of mutations in the *MYCN* gene (MA_n) and the genes encoding the MAPK/RAS pathway (ALK_n). $E_{MR,1}$ and $E_{MR,2}$ were obtained by calibration.

	$ALK_n = 0$	$ALK_n = 1$	$ALK_n = 2$
$MA_n = 0$	0.77*0.00	0.00 ($E_{MR,2}$)	0.00 ($E_{MR,2}$)
$MA_n = 1$	0.77*0.38	0.38 ($E_{MR,1}$)	0.38 ($E_{MR,1}$)

In supplementary figure 3E in [6], the mRNA expression level (after reversing the log transformation) of HBP1 associated with *MYCN* amplification is 25 % lower than that without. This translates to a 25 % increase in MYCN activity level. Therefore, if E_{MYCN} is 0.94 (found by calibration) when the *MYCN* gene and *ALK* gene are both amplified or activated, the corresponding value should be lower at 0.8*0.94 when only the *ALK* gene is amplified or activated. According to figure 1E in [6], a mutation in *ALK* causes a reduction of around 40 % in the mRNA expression level of HBP1. This translates to a 40 % increase in MYCN activity level. Therefore, if E_{MYCN} is 0.94 when the *MYCN* gene and *ALK* gene are both amplified or activated, the corresponding value should be lower at 0.71*0.94 when the *MYCN* gene is amplified but the *ALK* gene is neither amplified or activated. The combined effects of *MYCN* amplification and *ALK* amplification/activation are assumed to be multiplicative. The six values are presented in Table A.

In figure 2D in [7], the signature score of MAPK/RAS signalling associated with *MYCN* amplification and mutations in the genes encoding the MAPK/RAS signalling pathway is 0.4, while the score associated with only *MYCN* amplification is -0.38. On this basis, the two calibrated activity levels of the MAPK/RAS pathway ($E_{MR,1}$ and $E_{MR,2}$) were modified to estimate the remaining values in Table B.

E Collagen production

The production rate of collagen in a Schwann cell ($R_{collagen}$) was estimated by following this line of reasoning. A Schwann cell produces 0.3 ng of protein in 96 hours: 3.125e^{-12} g hour^{-1} [8]. In the cell, 12 % of protein synthesis contributes to collagen production [9]. The volume of hydrated collagen I is 1.89e^{12} um^3 g^{-1} [10]. Taken together, the matrix production rate due to one Schwann cell is $3.125e^{-12} \times 0.12 \times 1.89e^{12}$ or 0.70875 um^3 hour^{-1}.

References

[1] Del Monte U. Does the cell number 109 still really fit one gram of tumor tissue? Cell cycle. 2009;8(3):505-6.

[2] Hayflick L, Moorhead PS. The serial cultivation of human diploid cell strains. Experimental cell research. 1961;25(3):585-621.

[3] Warren DR, Partridge M. The role of necrosis, acute hypoxia and chronic hypoxia in 18F-FMISO PET image contrast: a computational modelling study. Physics in Medicine & Biology. 2016;61(24):8596.

[4] Grimes DR, Kelly C, Bloch K, Partridge M. A method for estimating the oxygen consumption rate in multicellular tumour spheroids. Journal of The Royal Society Interface. 2014;11(92):20131124.

[5] Ackermann S, Cartolano M, Hero B, Welte A, Kahlert Y, Roderwieser A, et al. A mechanistic classification of clinical phenotypes in neuroblastoma. Science. 2018;362(6419):1165-70.

[6] Claeys S, Denecker G, Durinck K, Decaesteker B, Mus LM, Loontiens S, et al. ALK positively regulates MYCN activity through repression of HBP1 expression. Oncogene. 2019;38(15):2690-705.

[7] Eleveld TF, Schild L, Koster J, Zwijnenburg DA, Alles LK, Ebus ME, et al. RAS–MAPK Pathway-Driven Tumor Progression Is Associated with Loss of CIC and Other Genomic Aberrations in NeuroblastomaNovel Mutations Drive the RAS–MAPK Pathway in Neuroblastoma. Cancer research. 2018;78(21):6297-307.

[8] Conlon I, Raff M. Differences in the way a mammalian cell and yeast cells coordinate cell growth and cell-cycle progression. Journal of biology. 2003;2:1-10.

[9] DeClerck YA, Bomann ET, Spengler BA, Biedler JL. Differential collagen biosynthesis by human neuroblastoma cell variants. Cancer research. 1987;47(24_Part_1):6505-10.

[10] Levick J. Flow through interstitium and other fibrous matrices. Quarterly Journal of Experimental Physiology: Translation and Integration. 1987;72(4):409-37.
