# Supplementary material for: Multicellular model of neuroblastoma proposes unconventional therapy based on multiple roles of p53
Source: PLoS Comput Biol. 2024 Dec 23;20(12):e1012648. doi: 10.1371/journal.pcbi.1012648 (PMC11723635; doi:10.1371/journal.pcbi.1012648)
Supplement: S3 Text — This text provides the details regarding each step of the simulation algorithm. (PDF) [file pcbi.1012648.s003.pdf]

S3 Text: Implementation of the Stochastic Simulation Process

Kenneth Y. Wertheim[1,2,3,4], Robert Chisholm[2], Paul Richmond[2], Dawn Walker[1,2]

[1]Insigneo Institute for *in Silico* Medicine, University of Sheffield, Sheffield, UK.

[2]Department of Computer Science, University of Sheffield, Sheffield, UK.

[3]Centre of Excellence for Data Science, Artificial Intelligence, and Modelling, University of Hull, Kingston upon Hull, UK.

[4]School of Computer Science, University of Hull, Kingston upon Hull, UK.

We devised a Monte Carlo algorithm to simulate how the multicellular model's variables change dynamically. After a user configures a simulation, a number of runs will be implemented automatically. A run starts with an initialisation routine before a pre-defined sequence of operations is iterated a finite number of times.

During configuration, the simulation requires six groups of inputs from the user.

1. The time scale is defined by its extent and grain. The simulation entails a positive integral number of time steps (N_{steps}) lasting T_{step} hours each. For example, if T_{step} is one hour, the first time step starts when t is zero and ends when t is one; t tracks the simulation's progress in hours. Considering the events making up the cell cycle are measured in hours, T_{step} is one hour by default. A standard schedule of induction chemotherapy comprises six to eight cycles of 21 days [1]. Therefore, N_{steps} is 3024 (18 weeks) by default. The spatial domain is a cube and the user must define its volume (V) and cellularity ($cellularity$).

2. The tumour's composition is defined by its histological type ($hist$) and grade of differentiation ($grade$). There are seven histological categories: neuroblastoma (zero), ganglioneuroblastoma (one), nodular ganglioneuroblastoma (two), intermixed ganglioneuroblastoma (three), ganglioneuroma (four), maturing ganglioneuroma (five), and mature ganglioneuroma (six) [2]. If $hist$ is one, it means that it is unclear whether the histological category is nodular or intermixed ganglioneuroblastoma. Therefore, $hist$ will be stochastically assigned to two or three. If $hist$ is four, it means that it is unclear whether the histological category is maturing ganglioneuroma or mature ganglioneuroma, so $hist$ will be stochastically assigned to five or six. An increase in $hist$ means more differentiated neuroblastoma cells and a more dominant Schwannian stromal component [2]. Regarding $grade$, the tumour is undifferentiated (zero), poorly differentiated (one), or differentiating (two) [2]. These parameters do not affect any state transition functions or other model components directly. They determine the simulation's initial state only, specifically the split between neuroblastoma and Schwann cell agents, and the neuroblastoma cell agents' degree of differentiation.

3. After creating the discrete neuroblastoma cell agents, their attributes must be initialised for each clone containing a subset of them. For each agent, in addition to the mutation vector, the user needs to specify whether ALT_n and $p53_n$ in the molecular vector are forcibly switched on and off respectively. The other attributes have default settings. For example, $N_{telo,n}$ is initialised according to the mutation vector and literature values [3]. Similarly, the Schwann cell agents have default attributes, so configuration is unnecessary.

4. The tumour's microenvironment has two aspects. First, the user needs to initialise C_{O2}. Second, the chemotherapy regimen is defined in terms of three vectors: $chemo_{start}$, $chemo_{end}$, and $chemo_{effects}$.

After configuration, the program creates an initial population of discrete autonomous agents. The initial number of agents is given by $V \times cellularity \times \rho$, where ρ is the number of cell agents in one µm^3 of volume not occupied by extracellular matrix. Together, $hist$ and $grade$ determine the initial split between neuroblastoma and Schwann cell agents in this population ($\theta_{sc,0}$), as well as the neuroblastoma cell agents' initial degree of differentiation ($deg_{diff,n}$ in the cellular vector). For example, if $hist$ and

grade are both zero, $\theta_{sc,0}$ is set stochastically between 0.05 and 0.17, and $deg_{diff,n}$ is set to zero. As *hist* and/or *grade* go up, $\theta_{sc,0}$ and $deg_{diff,n}$ go up too. The initial number of Schwann cell agents is therefore $V \times cellularity \times \rho \times \theta_{sc,0}$.

Next, as the initial distribution of extracellular matrix in the spatial domain is uniform, every voxel is assigned the same value for $M_{i,j,k}$: $1 - cellularity$.

The initialisation routine finishes by optimising the randomly assigned spatial coordinates of each agent by minimising the total overlap in the spatial domain with the centre-based mechanical model. The continuous automaton is set up based on the optimised spatial coordinates. The vasculature is established by initialising R_{O2} and N_{ang} with the *Vasculature* function.

The algorithm iterates the following operations N_{steps} times.

1. The neuroblastoma cell agents are sequentially evaluated. Each agent senses the tumour microenvironment and the stimuli from the other agents (*Sense* function) and then attempts to progress through the cell cycle (*Cycle* function). An apoptotic or necrotic agent may get removed by the *Remove* function, which mimics the action of immune cells [4, 5]. A living agent may divide to produce two daughter cell agents (*Divide* function).

2. The Schwann cell agents are evaluated one by one by following the same steps as in the first operation. However, gene expression is not considered in this operation and the cellular phenomena are not regulated by any gene products (they have none). Furthermore, these agents do not differentiate.

3. The total cell-cell overlap in the spatial domain is minimised using the centre-based mechanical model, thereby updating the agents' spatial coordinates. It involves updating the continuous automaton in terms of the spatial distributions of various cell populations too, including the addition of new voxels if the agents are beyond the outer boundary.

4. The number of VEGF-producing neuroblastoma cell agents and the number of living Schwann cell agents are used to update the progress of angiogenesis and hence the vasculature (R_{O2}).

5. The oxygen level, C_{O2}, is updated according to the balance between R_{O2} and oxygen consumption. In addition, this step updates $M_{i,j,k}$ by considering the number of collagen-producing Schwann cell agents in each voxel; the model assumes that contact inhibition arrests collagen production.

References

[1] Habib EE, El-Kashef AT, Fahmy ES. Management of neuroblastoma: a study of first-and second-line chemotherapy responses, a single institution experience. Oncology Reviews. 2012;6(1).

[2] Shimada H, Ambros IM, Dehner LP, Hata Ji, Joshi VV, Roald B. Terminology and morphologic criteria of neuroblastic tumors: recommendations by the International Neuroblastoma Pathology Committee. Cancer: Interdisciplinary International Journal of the American Cancer Society. 1999;86(2):349-63.

[3] Ackermann S, Cartolano M, Hero B, Welte A, Kahlert Y, Roderwieser A, et al. A mechanistic classification of clinical phenotypes in neuroblastoma. Science. 2018;362(6419):1165-70.

[4] Dunster JL, Byrne HM, King JR. The resolution of inflammation: a mathematical model of neutrophil and macrophage interactions. Bulletin of mathematical biology. 2014;76(8):1953-80.

[5] Song P, An J, Zou MH. Immune clearance of senescent cells to combat ageing and chronic diseases. Cells. 2020;9(3):671.
