# Supplementary material for: Multicellular model of neuroblastoma proposes unconventional therapy based on multiple roles of p53
Source: PLoS Comput Biol. 2024 Dec 23;20(12):e1012648. doi: 10.1371/journal.pcbi.1012648 (PMC11723635; doi:10.1371/journal.pcbi.1012648)
Supplement: S4 Text — This text explains how we fixed the unconstrained parameters. (PDF) [file pcbi.1012648.s004.pdf]

S4 Text: Calibration

Kenneth Y. Wertheim[1,2,3,4], Robert Chisholm[2], Paul Richmond[2], Dawn Walker[1,2]

[1]Insigneo Institute for *in Silico* Medicine, University of Sheffield, Sheffield, UK.
[2]Department of Computer Science, University of Sheffield, Sheffield, UK.
[3]Centre of Excellence for Data Science, Artificial Intelligence, and Modelling, University of Hull, Kingston upon Hull, UK.
[4]School of Computer Science, University of Hull, Kingston upon Hull, UK.

First, the mechanical model was calibrated. Contact inhibition stops a living cell from progressing in the cell cycle and in the case of a living Schwann cell, stops it from secreting extracellular matrix. A heuristic was developed to ensure that matrix production does not continue in a virtual tumour when it runs out of space. In this test, parameters and initial conditions aligning with a highly malignant case were used. For example, the entire mutation vector was switched on and E_{MYCN}, $E_{MR,1}$, and $E_{MR,2}$ were all set to one (maximum). Chemotherapy was switched off too. Based on reasonable assumptions about cell sizes and volume expansion (up to 100 % in 3024 hours), an optimal value (18.48 µm) was found for L_{nghbr}. In the computational experiment, this parametric value limited the maximum number of living agents to under 3.6 times the corresponding initial number, in agreement with our assumption about volume expansion. Using this value ($L_{nghbr} = 18.48$ µm), the entire experiment was repeated using different values of V, increasing from 2e^6 µm^3 (original volume) to 2.56e^8 µm^3 (maximum volume) gradually. Higher expansion ratios were observed in the larger virtual tumours in this cohort. One plausible explanation is that the agents in their smaller counterparts resolved cell-cell overlap more effectively. To encourage cell cycling, L_{nghbr} was reduced gradually. By trial and error, 17.33 µm was found to be better. During each experiment, the maximum displacement experienced by an agent in one force resolution step (Δt) was recorded. The maximum displacement experienced by the same agent in one agent time step (T_{step}) was recorded too. The former was found to be less than 10 % of $\frac{L_{cell}}{2}$ in all experiments; the latter, less than L_{cell}. These results indicate that the chosen parametric values allow smooth cellular migration.

Without considering the exact effects of chemotherapy on individual gene products (it induced apoptosis directly in this set of simulations), we calibrated the fitting parameters collectively in a tournament-style pipeline. Overall, we used the Latin hypercube sampling technique to generate 3000 near-random combinations of fitting parameters and passed them through six elimination rounds. In each round, we used the remaining parameters to reproduce a dataset found in the literature. The rounds were arranged in an increasing order of sophistication, with the first dataset—*in vitro* and without genomic data—being the coarsest and the sixth—clinical, patient-specific, and with known mutations—being the most precise.

1. We attempted to reproduce the growth kinetics of neuroblastoma observed in an *in vitro* study [1]. For each combination, we calculated the residual sum of squares of the differences between the simulation and experimental results. In this round, every virtual tumour was assigned a random combination of mutations since the real tumours used in the *in vitro* study were not differentiated on this basis. The 1000 parametric combinations that gave the smallest residual sums entered the second round.

2. We attempted to reproduce the hypoxic response of neuroblastoma observed in another *in vitro* study [2]. As in the first round, random combinations of mutations were used in the simulations. For each parametric combination, we squared the difference between the simulated and observed final population sizes. This metric and the one from the first round were considered together to find the top 50 combinations.

3. These 50 combinations were used to simulate the dynamics between neuroblastoma and Schwann cells. Using each combination, we simulated the dynamics of each population in isolation and in conjunction, with and without *MYCN* amplification. In each simulation, the cycling proportion

of neuroblastoma cells, the cycling proportion of Schwann cells, and the apoptotic proportion of neuroblastoma cells were recorded at selected time points. The results were compared to the experimental data from an *in vitro* study [3], resulting in three metrics. Based on the metrics, we shortlisted 10 parametric combinations for the fourth round.

4. Then, we considered the relationship between the histology of a tumour and the clinical outcome. We attempted to reproduce the clinical outcomes—progression, regression, or differentiation—of real intermixed ganglioneuroblastoma and maturing ganglioneuroma patients [4]. When a real tumour under consideration had its *MYCN* amplified, chemotherapy was simulated in a non-specific manner: it induced apoptosis directly instead of inhibiting gene products. Four of the remaining combinations parameterised realistic simulations.

5. This round was designed on the basis of a more recent clinical study [5]. Using each of the four remaining parametric combinations, we tried to reproduce the clinical outcomes of 10 patient groups with different mutations. After considering the simulation results holistically, we eliminated one more parametric combination, leaving three for the final round.

6. The final round was designed based on the same clinical study [5], but we used a different dataset, one about *MYCN*-amplified patients only, to identify the best parametric combination.

After the tournament, as additional information was made available by our PRIMAGE partners, we continuously refined selected parameters to render the multicellular model suitable for simulating *in vivo* and clinical scenarios.

1. We simulated a tumour's response to chemotherapy inhibiting its CHK1, JAB1, HIF, MYCN, telomerase, and p53, as well as damaging its DNA. Unlike the simulations carried out in the tournament, chemotherapy was not allowed to induce apoptosis directly in this study. We refined two parameters describing cellular responses to chemotherapy and DNA impairment—$P_{DNA,c}$ and P_{apop}—by changing their values—aided by Latin hypercube sampling—to reproduce the extent of tumour shrinkage in a real patient encoded as B29FF3BE within the PRIMAGE project [6]. Separately, we decided to increase the initial telomere lengths to reduce the transient noises at the beginning of a simulation.

2. As mostly *in vitro* data were used in the tournament, selected parameters controlling cell cycling and non-mechanical cell-cell interactions—$P_{cycle,nb}$, $P_{cycle,sc}$, $R^{jux}_{pro,sc}$, $R^{jux}_{diff,nb}$, R_{diff}, and $R^{jux}_{apop,nb}$—were refined for *in vivo* use. Informed by literature values found in several sources [7–9], we set constraints on the population's doubling time, the growth rate of neuroblastoma cells, and the growth rate of Schwann cells. By a combination of Latin hypercube sampling and *ad hoc* exploration, we refined these parameters before repeating the first step to calibrate $P_{DNA,c}$ and P_{apop} again.

3. Next, we turned our attention to differentiation. By a combination of Latin hypercube sampling and *ad hoc* exploration, we refined $P_{cycle,nb}$, $P_{cycle,sc}$, $R^{jux}_{diff,nb}$, and R_{diff} further to ensure that a virtual tumour belonging to the histological category of neuroblastoma cannot differentiate to healthy neurons in a simulation.

4. By Latin hypercube sampling, we refined $P_{DNA,c}$ and P_{apop} even further with respect to an even more precise chemotherapy regimen: the rapid COJEC regimen, which inhibits a tumour's JAB1, MYCN, and telomerase only [10, 11]. The extent of tumour shrinkage in patient B29FF3BE was used as the benchmark again.

References

[1] Tumilowicz JJ, Nichols WW, Cholon JJ, Greene AE. Definition of a continuous human cell line derived from neuroblastoma. Cancer research. 1970;30(8):2110-8.

[2] Warren DR, Partridge M. The role of necrosis, acute hypoxia and chronic hypoxia in 18F-FMISO PET image contrast: a computational modelling study. Physics in Medicine & Biology. 2016;61(24):8596.

[3] Ambros IM, Attarbaschi A, Rumpler S, Luegmayr A, Turkof E, Gadner H, et al. Neuroblastoma cells provoke Schwann cell proliferation in vitro. Medical and Pediatric Oncology: The Official Journal of SIOP—International Society of Pediatric Oncology (Societé Internationale d'Oncologie Pédiatrique). 2001;36(1):163-8.

[4] Okamatsu C, London WB, Naranjo A, Hogarty MD, Gastier-Foster JM, Look AT, et al. Clinicopathological characteristics of ganglioneuroma and ganglioneuroblastoma: a report from the CCG and COG. Pediatric blood & cancer. 2009;53(4):563-9.

[5] Ackermann S, Cartolano M, Hero B, Welte A, Kahlert Y, Roderwieser A, et al. A mechanistic classification of clinical phenotypes in neuroblastoma. Science. 2018;362(6419):1165-70.

[6] Martí-Bonmatí L, Alberich-Bayarri Á, Ladenstein R, Blanquer I, Segrelles JD, Cerdá-Alberich L, et al. PRIMAGE project: predictive in silico multiscale analytics to support childhood cancer personalised evaluation empowered by imaging biomarkers. European radiology experimental. 2020;4(1):1-11.

[7] Houghton J, Taylor D. Growth characteristics of human colorectal tumours during serial passage in immune-deprived mice. British Journal of Cancer. 1978;37(2):213-23.

[8] George BA, Yanik G, Wells RJ, Martin LW, Soukup S, Ballard ET, et al. Growth patterns of human neuroblastoma xenografts and their relationship to treatment outcome. Cancer. 1993;72(11):3331-9.

[9] Steel G. Growth kinetics of tumours: cell population kinetics in relation to the growth and treatment of cancer. 1977: Oxford University Press. USA;.

[10] Garaventa A, Poetschger U, Valteau-Couanet D, Luksch R, Castel V, Elliott M, et al. Randomized Trial of Two Induction Therapy Regimens for High-Risk Neuroblastoma: HR-NBL1.5 International Society of Pediatric Oncology European Neuroblastoma Group Study, Journal of Clinical Oncology. Journal of Clinical Oncology. 2021;39(23):2552-63.

[11] Smith V, Foster J. High-risk neuroblastoma treatment review. Children. 2018;5(9):114.
