# Supplementary material for: Multicellular model of neuroblastoma proposes unconventional therapy based on multiple roles of p53
Source: PLoS Comput Biol. 2024 Dec 23;20(12):e1012648. doi: 10.1371/journal.pcbi.1012648 (PMC11723635; doi:10.1371/journal.pcbi.1012648)
Supplement: Attachment — Submitted filename: Response_letter.pdf [file pcbi.1012648.s005.pdf]

University of Sheffield | School of Computer Science

Professor Heidi Christensen
Head of School

Regent Court
211 Portobello
Sheffield
S1 4DP

Telephone: +44 (0) 114 222 1800
Email: dcs@sheffield.ac.uk

1st October 2024

Manuscript number: **PCOMPBIOL-D-24-00310**

Dear Dr Meier-Schellersheim,

Thank you very much for the editorial and independent reviews. We are glad that you are likely to accept the manuscript for publication. Furthermore, we are grateful for the positive comments and constructive feedback provided by the reviewer and the time they have spent reviewing our manuscript in considerable detail.

Based on their recommendations, we have updated the manuscript. In the following paragraphs, we will provide our responses to their comments and describe how the updated manuscript is different from and better than the original manuscript.

`Reviewer #1: Your study introduces an impressive multicellular model of neuroblastoma, delving into the dynamics of MYCN amplification clones and exploring various chemotherapy combinations. Moreover, your efforts to validate the model's capacity to reproduce established literature findings and underscore its potential for generating digital replicas of neuroblastoma patients are admirable. Below, I have outlined some comments and suggestions aimed at further enhancing the clarity, rigor, and impact of your manuscript:'

We thank the reviewer for reading our work and their kind words.

`1 - In addressing the abstract and specific parts of the text, the description of the major components of the multicellular model of neuroblastoma could benefit from refinement.

Emphasizing the primary features, such as the continuous automaton and the center-based agent-based model, would enhance clarity and conciseness in conveying the key aspects of the model.’

The reviewer is correct. The three major components are described in detail in the updated abstract and the beginning of section 3, as well as the beginning of section 6. They are also named at the end of section 2.

`2 - In certain sections of the manuscript, the model is referred to as the first computational model of neuroblastoma. However, it is important to note that this is not the initial model constructed, as portions of this model have previously been published by the authors in the article cited: https://doi.org/10.1016/j.cmpb.2023.107742. Clarifying this point would help ensure accuracy in the description of the model's novelty and previous iterations.’

We agree with the reviewer about the importance of clarity. The new author summary clarifies that we built, to the best of our knowledge, the first multicellular computational model during the PRIMAGE project, but a portion of our work relating to this model is already in print. This point is made with references at the end of section 2 and reinforced at the start of section 5. This manuscript is the first detailed and fully complete description of the multicellular model.

`3- To enhance clarity and facilitate better referencing in the explanation, it is suggested to break down Figure 1a into subfigures. Additionally, it appears that part of the figure, specifically the green fluorescent marker cells, lacks explanation, and it seems disconnected from the model structure.’

Again, we agree with the reviewer about the importance of clarity. The updated figure 1 contains five subfigures, each of which describes a specific model component. The first paragraph of the updated section 3 references the subfigures one by one as it describes the model structure. Indeed, the green fluorescent marker cells in the original Figure 1a are there for illustration only. They do not represent neuroblastoma or Schwann cells. The updated figure 1 does not contain these misleading marker cells.

`4- Consider rearranging Figure 3 to improve readability, particularly in areas where the letters are too small.’

We believe that figure 3 is an effective visual message about the calibration pipeline, but we agree with the reviewer that some of the words are too small. Table 1 in the updated manuscript presents the same message in bigger words to complement figure 3.

`5- In line 146, the statement mentions, "An ensemble was categorised only if the majority of its runs had ended in one of the three outcomes." It is important to address the uncertainty surrounding this classification and whether it converges over repeated runs. Additionally, clarification is needed regarding how many ensembles have unique classifications and how the outcomes of the ensemble change in accordance with this hypothesis. Furthermore, there is a lack of commentary on why no differentiation occurs and whether any run replicate consistently yields this outcome. These observations are significant, particularly in the context of precision medicine.'

We thank for the reviewer for their advice. In 1154 ensembles, every run produced a progressing tumour. In 42 ensembles, every run produced a regressed tumour. The remaining four ensembles were found to contain runs producing one progressing and nine regressed tumours; three progressing and seven regressed tumours; one progressing and nine regressed tumours; and six progressing and four regressed tumours. Overall, all but nine out of 12000 runs converged to their ensemble outcomes. Based on the results, we argue that the level of uncertainty is low.

We agree that the classification thresholds collectively represent a mere hypothesis. The high number (1196 out of 1200) of ensembles with unique classifications indicates that the ensemble outcomes are robust to changes in the hypothesis.

None of the runs produced a differentiated tumour. This result is both unsurprising and reassuring. As we were interested in malignant tumours, we did not evaluate any virtual tumours with more Schwann cell agents than neuroblastoma cell agents. That differentiation did not occur as an emergent phenomenon during the simulations is consistent with the consensus that spontaneous regression or differentiation typically occurs in benign cases.

These additional details are in the updated subsection 3.1.

`6- In line 151, it is stated that "simulations. Figure 4(b) indicates that, as expected, hypoxia led to regression. On the other hand, the initial clonal composition of a virtual tumor had very little impact on its ensemble outcome (figures 5 and 6)." While this observation is notable, incorporating statistical tests into the figures would provide a more scientifically rigorous analysis rather than relying solely on visual inspection. Additionally, comparing outcomes by medians at the end of this paragraph may not be sufficient to determine if there are any significant variations. Therefore, a more comprehensive statistical analysis is warranted.'

We agree that visual inspection and point estimates of medians are not sufficient for a scientifically rigorous analysis. We conducted a more comprehensive statistical analysis to back up our observation.

For each subclone, we resampled from its 1200 initial fractional compositions to build two confidence intervals. First, we resampled the sample 12000 times, taking 45 values with replacement and recording their medium on each occasion. Using the 12000 mediums, we built a 95 % confidence interval for the subclone's medium initial fractional composition. Second, we repeated the procedures with a resample size of 1155 values to build another 95 % confidence interval.

In the updated manuscript, figure 7 compares each subclone's medium initial fractional composition in the 45 virtual tumours that ended in regression with the first confidence interval built for it and the medium in the 1155 virtual tumours that ended in progression with the second confidence interval. Only one point estimate of a medium (subclone four in the regressed virtual tumours) falls outside its confidence interval. Considering the probability that a result that extreme occurs by chance is 5 % and we calculated 48 confidence intervals, it is reasonable to dismiss that point estimate as a chance event rather than a significant variation. We conclude that the model's ensemble outcome is generally not very sensitive to its initial clonal composition.

The updated subsection 3.1 presents this more rigorous analysis.

`7- In line 201, the statement mentions, "It is also clear that one expression level (MR1) of the MAPK/RAS signaling pathway is upregulated relative to the other (MR0). However, MR1 was set to be higher than MR0 to reflect the influence of MYCN amplification on the pathway, so the difference is trivial." This sentence sounds more like a hypothesis than a result. Additionally, if the difference is considered trivial, it may not be necessary to include it in the results.’

The reviewer is spot on. The difference is a hypothesis, not a result. The statement is in this section to caution the reader against treating it as a result. The corresponding statement in the updated manuscript (line 249) makes this clear.

`8- In line 204, it is stated, "Performing the same analysis on the 870 progressing virtual tumors' profiles gave us a first principal component responsible for 20% of the variance in the larger dataset (not shown)." If the information is not shown, it should not be included in the main text unless it is provided in the Supplementary Material. Additionally, at the end of this paragraph, it is asserted that there is not a latent feature. However, this conclusion requires further scientific evidence. Specifically, it is necessary to define what constitutes a small difference (as "small" is relative) and what qualifies as a latent feature in this context.’

We have implemented the suggested changes.

In the updated subsection 3.2, the information (larger dataset) is not mentioned. Instead, the first principal component extracted from the 283 profiles is compared to a hypothetical observable with respect to white noise, in terms of how explanatory they are in the two scenarios.

Contrary to what the original manuscript says, the dataset contains latent features, which are underlying variables that are not directly recorded in the dataset but can be inferred from it to explain the variance therein. However, even the best latent feature tells us less than 1.5 times more about the dataset than an arbitrary observable does about white noises. The weak latent features indicate that the six observables (gene expression levels) influence a *MYCN*-amplified clone independently. In the updated subsection 3.2, what constitutes a latent feature is defined and the correct explanation is provided.

`9- In line 218, it is stated that "On average (10 runs), the wild-type, MYCN-amplified, TERT-rearranged, and ATRX-inactivated clones contained approximately 2.63 × 10^5 (261873–265109), 2.68 × 10^5 (266207–269300), 2.63 × 10^5 (261921–265248), and 2.63 × 10^5 (261800–264824) living neuroblastoma cell agents when this simulation finished." To strengthen the analysis, a statistical test of these populations could confirm whether they appear identical, and whether any differences are statistically significant rather than merely minute in relative terms. Additionally, the assertion of consistency lacks scientific grounding; it would be beneficial to support this claim with evidence from the literature or provide an explanation for the potential consistency observed.'

Inspired by the reviewer's feedback, we conducted a series of one-tailed paired sample t-tests, as well as an exact permutation test and an F-test for ANOVA. The p-values indicate that the *MYCN*-amplified and *TERT*-rearranged clones did outgrow the wild-type clone on average, but the hypothesis that the *ATRX*-inactivated clone did is statistically insignificant. Furthermore, the p-values from the two ANOVA tests support the hypothesis that the three mutated clones achieved statistically different outcomes in the simulation. The procedures are explained in detail in the updated subsection 6.7. The results are presented in the updated subsection 3.2.

The original manuscript does provide scientific grounding for the assertion of consistency in subsection 6.7. In the updated subsection 3.2, there is a cross-reference to clarify this point.

`10- In line 241, it is stated, "Working with 96 datasets comprising 100 final subclone fractions each, we resampled each dataset 100 times, taking 10 samples with replacement and averaging them arithmetically to obtain a sample statistic every time, leading to 100 sample statistics per

dataset." However, there is a lack of description regarding the specific nature of the 96 datasets being referenced.'

We apologise for the confusion. There is such a description in subsection 6.7. In the updated subsection 3.3, the description is reproduced.

`11- In the Materials and Methods section, it's important to include a summary that provides an overall description of the integration of methods, including the model components such as the continuous automaton, autonomous discrete agents, and mechanical model. This summary will help readers understand how these methods are combined and utilized in the study's approach.'

The new Materials and Methods section contains the summary recommended by the reviewer. It is at the start and right before subsection 6.1.

`12- In line 634, the statement "This simplified representation of the microenvironment, especially the assumption of homogeneity, is justified as the model only describes a small region in a tumor" lacks clarification on the specific size of the region for which the hypothesis of homogeneity is valid. A literature review on the topic could provide insights into the appropriate scale at which homogeneity assumptions hold true in tumor microenvironments.'

We thank the reviewer for alerting us to an error of communication. That assumption is only justified in a voxel whose side spans 30 microns, which is significantly smaller than the diffusion distance of oxygen in tumour tissues (typically between 100 and 150 microns). The spatial domain contains many voxels and spans 2000 microns in each direction, so the assumption of homogeneity is not valid.

However, within the multi-scale orchestrated computational framework encompassing the multicellular model (https://doi.org/10.1016/j.cmpb.2023.107742), vascularisation and cellularity data extracted from dynamic contrast-enhanced magnetic resonance imaging (DCE-MRI) and diffusion-weighted imaging (DWI) are applied to the whole tumour–level transport model, so heterogeneity is already handled at a higher spatial scale. We decided to assume homogeneity at the tissue scale for this reason.

The updated subsection 6.1 does not include the incorrect statement. Instead, it acknowledges that the assumption of homogeneity is not ideal, but tumour heterogeneity is described at a higher spatial scale within the broader context of the PRIMAGE project. In the updated subsection 4.5, a spatially heterogeneous vasculature coupled with a set of reaction-diffusion-advection equations is listed as a future addition.

`13- In line 684, the statement mentions, "If it is apoptotic or necrotic, it may be removed from the system (imitation of how immune cells act)." To clarify the observation within the parenthesis regarding how this imitation occurs, it would be helpful to provide specific details or mechanisms through which the removal of apoptotic or necrotic cells from the system is simulated in the model to mimic the behavior of immune cells.'

The updated paragraph containing that statement explains that the stated removal process is stochastic and occurs according to the probability `P_{lysis}'. It explains that removal means the space occupied by a dead cell agent becomes available to its neighbours. Furthermore, it is acknowledged in the discussion (subsection 4.3.2) that this is a highly simplified way of imitating the immune system, but since detailed models of the human immune system have been and are being developed by other people, its simplistic setup will facilitate model integration by limiting compatibility issues.

`14- In the Mechanical Model section (6.3), including a mention that the off-lattice model is a center-based model would enhance clarity and facilitate understanding for readers. Additionally, at the end of this section, it is stated, "Empirical tests were conducted..., which was judged to be physically realistic." Providing more details about the criteria used to judge the physical realism of the model, such as the magnitude of speed and typical cellular radius, would offer a more comprehensive understanding of the evaluation process.'

The statement that the mechanical model is a centre-based model is at the beginning of the updated subsection 6.3. At the end, literature values are provided to assure the reader of the model's physical realism.

`15- In section 6.4.1, it would be beneficial to enhance clarity by listing the inputs required for the simulation before directing the reader to other paragraphs where these inputs are described. This approach can help readers understand the key components of the simulation process more effectively.'

We agree with the reviewer's point of view. The updated subsection 6.4.1 begins with a list enumerating the required inputs.

`16- In line 738, it is suggested to remove the sentence "which has a smaller fraction of Schwann cell agents and less differentiated neuroblastoma cell agents than ganglioneuroma," as it appears to be duplicative.'

The quoted sentence describes the relationship between ganglioneuroblastoma and ganglioneuroma, but it is a bit convoluted. In the corrected manuscript, the updated sentence simply states that ganglioneuroblastoma and ganglioneuroma have the same relationship as neuroblastoma and ganglioneuroblastoma do.

`17- In line 740, the phrase "our desire to use the whole range of each variable" does not provide a scientific rationale for the exploration. Consider revising this statement to reflect a more scientifically grounded motivation for the approach taken in the study.'

The reviewer is right about the importance of explaining things clearly. The updated manuscript does not contain the quoted phrase. Furthermore, subsection 6.4.1 in the updated manuscript makes our rationale and assumptions explicit. The relevant part is in the third paragraph.

`18- In line 744, it's necessary to define the parameter "deg_{diff,n}" and clarify how it is stochastically determined, including the distribution used. Similarly, this information should also be provided in lines 754, 759, and 762.'

The technical details requested by the reviewer are indeed essential for reproducing the study. In the updated manuscript, this variable is defined as the extent to which a neuroblastoma cell agent is differentiated along the neural crest–derived sympathoadrenal lineage. The definition is supported by two references. Furthermore, the fact that its value is sampled by the program from the continuous uniform distribution is stated clearly.

`19- In the model calibration section, it's essential to provide details on how the 22 parameters were chosen and the selection ranges for each parameter. This information is crucial for understanding the calibration process and ensuring transparency in the model development.'

We apologise for the confusion. We did not actively select these 22 normalised parameters. They just emerged by a process of elimination because we failed to find literature values for them. Since they were entirely unconstrained at the start of the calibration stage, each of them was sampled from the entire range between zero and one. This information is provided immediately after the list of unconstrained parameters in subsection 6.5 in the updated manuscript.

`20- In some calibration studies, additional details are needed regarding the problem statement, including the target and metrics used in the calibration process.'

In response to the reviewer's comment, we added the requested details to the original manuscript to prepare the updated manuscript. The requested details are in the updated section 6.5.

`a) In the first round, clarification is needed on how a quantity representing the growth kinetics was defined and identified as the target.'

As reported in chart 1 of the cited paper (PMID: 5459762), the number of viable cells in their culture was recorded at the start of the experiment, and after 24, 48, 72, 96, and 168 hours. In the first calibration study, the ratio between the number of living cell agents (neuroblastoma and Schwann cells) and the initial number of living cell agents was recorded at the beginning, and after 24, 48, 72, 96, and 168 time steps. For each parametric combination, the six pairs of simulated and experimental cell counts were squared independently and then summed to give the metric of this study. These additional details are in the updated subsection 6.5.

`b) In the fourth round, it's necessary to define how the histology of the tumor and clinical outcomes were determined and utilized to select the four candidates from the initial ten. In the main manuscript, it is mentioned that this selection was based on the cited "realist probabilities" in line 837, but further clarification is needed on the meaning of this term and how it was quantified.'

Table V of the cited paper (PMID: 19530234) reports the clinical outcomes of patients whose histological categories were intermixed ganglioneuroblastoma or maturing ganglioneuroma. In our simulations, we mimicked a histological category by initialising the split between neuroblastoma cell and Schwann cell agents, as well as the neuroblastoma cell agents' degree of differentiation. If a run finished without any living neuroblastoma cells, it was considered a regressed tumour. If a run finished with just living neuroblastoma cells that were at least 90 % differentiated on average, it was considered a differentiated tumour. If a run finished with just living neuroblastoma cells that were less than 90 % differentiated on average, it was considered a progressing tumour. Only cell counts, not tumour volumes, were considered in this classification step. In table V of the cited paper, the five-year event-free survival rate of the intermixed ganglioneuroblastoma patients is reported to be 94.1 %. Six of the 10 parametric combinations led to progression in at least 64 % of the runs simulating intermixed ganglioneuroblastoma patients, so they were eliminated. The remaining four led to regression or differentiation in 100 % of the runs. The five-year event-free survival rate of the maturing ganglioneuroma patients is reported to be 100 %. The remaining four parametric combinations gave us simulation results in agreement with this clinical observation too. Since they gave us realistic probabilities, we kept them for the next round. These additional details are in the updated subsection 6.5.

`c) In the fifth and sixth rounds, information is missing about the metrics and targets used to select three from four candidates, as well as in the sixth round for selecting one from four candidates.'

Round five. Supplementary figures S16A and S16B of the cited paper (PMID: 30523111) report the clinical outcomes of 43 patients. We combined them to form 10 patient groups based on their mutation profiles and how they were treated. After applying a group's mutation profile to the multicellular model, we simulated its dynamics. Similar to the fourth round, we classified the outcome of a run as regression, differentiation, or progression. One parametric combination consistently failed to produce a progressing tumour when it was expected, so it was eliminated.

Round six. Supplementary figure S18A of the cited paper (PMID: 30523111) reports the clinical outcomes of 49 patients with *MYCN*-amplified tumours. We combined them to form four patient groups based on their mutation profiles. One of the three remaining parametric combinations gave us more realistic simulation results (around half of the runs ended in progression as expected) than the other two when *p53* inactivation was not applied to the model, so it was chosen as the tournament winner.

These additional details are in the updated subsection 6.5.

`Providing these details will enhance transparency and understanding of the calibration process. For the other rounds, the supplemental material has a satisfactory description.'

We hope that the updated subsection 6.5 is sufficiently transparent and informative.

`21 - In section 6.6.1:
a) It could be beneficial to define the population structure instead of requesting the reader to look at Figure 1, which may be distant from the current text.'

The reviewer is correct. The definition is reproduced in the updated subsection 6.6.1.

`b) Be more specific about what is meant by "determined randomly," including clarification on the distribution used (uniform, normal, etc.).'

We apologise for the confusion. A uniform distribution over the 24 clone IDs was used. The updated subsection 6.6.1 contains this clarification.

`c) In the definition of the range of oxygen level, provide an explanation if these values have units associated with them.'

The updated subsection 6.6.1 contains this explanation.

We hope that the revised manuscript is acceptable for publication. As requested, submitted with this letter are two versions: one with highlights and a clean version, in addition to revised figure files. Please do not hesitate to contact us if anything further is required.

Yours sincerely,
Dr Kenneth Y. Wertheim
Dr Dawn Walker
